# Lon upregulation contributes to cisplatin resistance by triggering NCLX-mediated mitochondrial Ca^2+^ release in cancer cells

**DOI:** 10.1038/s41419-022-04668-1

**Published:** 2022-03-16

**Authors:** Vidhya Tangeda, Yu Kang Lo, Ananth Ponneri Babuharisankar, Han-Yu Chou, Cheng-Liang Kuo, Yung-Hsi Kao, Alan Yueh-Luen Lee, Jang-Yang Chang

**Affiliations:** 1 Joint PhD program in molecular medicine, National Central University & National Health Research Institutes, Zhunan, Miaoli, 35053 Taiwan; 2 National Institute of Cancer Research, National Health Research Institutes, Zhunan, Miaoli 35053 Taiwan; 3 Department of Life Sciences, College of Health Sciences & Technology, National Central University, Taoyuan, Zhongli 32001 Taiwan; 4 Department of Biotechnology, College of Life Science, Kaohsiung Medical University, Kaohsiung, 80708 Taiwan; 5 Institute of Biotechnology and Pharmaceutical Research, National Health Research Institutes, Zhunan, Miaoli 35053 Taiwan

**Keywords:** Cancer therapeutic resistance, Mitochondria, Calcium and vitamin D

## Abstract

Mitochondria are the major organelles in sensing cellular stress and inducing the response for cell survival. Mitochondrial Lon has been identified as an important stress protein involved in regulating proliferation, metastasis, and apoptosis in cancer cells. However, the mechanism of retrograde signaling by Lon on mitochondrial DNA (mtDNA) damage remains to be elucidated. Here we report the role of Lon in the response to cisplatin-induced mtDNA damage and oxidative stress, which confers cancer cells on cisplatin resistance via modulating calcium levels in mitochondria and cytosol. First, we found that cisplatin treatment on oral cancer cells caused oxidative damage of mtDNA and induced Lon expression. Lon overexpression in cancer cells decreased while Lon knockdown sensitized the cytotoxicity towards cisplatin treatment. We further identified that cisplatin-induced Lon activates the PYK2-SRC-STAT3 pathway to stimulate Bcl-2 and IL-6 expression, leading to the cytotoxicity resistance to cisplatin. Intriguingly, we found that activation of this pathway is through an increase of intracellular calcium (Ca^2+^) via NCLX, a mitochondrial Na^+^/Ca^2+^ exchanger. We then verified that NCLX expression is dependent on Lon levels; Lon interacts with and activates NCLX activity. NCLX inhibition increased the level of mitochondrial calcium and sensitized the cytotoxicity to cisplatin in vitro and in vivo. In summary, mitochondrial Lon-induced cisplatin resistance is mediated by calcium release into cytosol through NCLX, which activates calcium-dependent PYK2-SRC-STAT3-IL-6 pathway. Thus, our work uncovers the novel retrograde signaling by mitochondrial Lon on resistance to cisplatin-induced mtDNA stress, indicating the potential use of Lon and NCLX inhibitors for better clinical outcomes in chemoresistant cancer patients.

## Introduction

Mitochondria are critical organelles that determine cell fate through their multiple cellular functions, including energy production, free radical production, calcium signaling, autophagy, and programmed cell death (apoptosis). Mitochondria are also considered as important organelles to response cellular stress [1]. Mitochondrial Lon is a multiple-function protein that has proteolytic [2], chaperone [3–5], and DNA-binding activity [6, 7], which plays a crucial role in protein quality control and maintains the normal function and homeostasis of mitochondria [2, 8]. Mitochondrial Lon is a stress protein and induced by various stresses, such as unfolded protein response, hypoxia, and oxidative stress [9, 10]. Upon oxidative stress, Lon upregulation is required for cancer cell survival and tumorigenesis through p38-NF-κB-dependent pathways [9, 11, 12]. Mitochondria act as a major source of endogenous ROS that escape from the electron transport chain [13, 14]. We found that mitochondrial ROS generation induced by Lon is involved in the function of Complex I, NADH dehydrogenase, and PYCR1 enzyme [9, 12]. Mitochondrial ROS causes direct damages to metabolic enzymes, lipids, and mitochondrial DNA (mtDNA) [15, 16]. Although mitochondrial Lon showed a reduced binding ability to mtDNA upon oxidative stress [17], the underlying mechanism of retrograde signaling by Lon on mtDNA damage remains to be fully elucidated.

Cisplatin is a first-line chemotherapeutic drug used for the treatment of head and neck cancers but chemoresistance remains a persistent problem [18]. Cisplatin treatment directly damages mtDNA and induces oxidative stress, thereby leading to mitochondrial dysfunction [19]. Several studies showed that mitochondria-induced oxidative stress increases the cisplatin resistance by metabolic reprogramming [20, 21], escapes from cell death by activating stress response pathways [9, 22], and activates migration and epithelial-mesenchymal transition (EMT) of cancer cells [12]. However, the role of mitochondrial Lon in the cisplatin-induced oxidative stress and resistance is yet unknown.

Calcium (Ca^2+^) homeostasis in mitochondria is an important phenomenon to maintain cell metabolism and viability. Mitochondria buffer calcium to modulate intracellular signals via calcium influx and efflux channels [23]. Mitochondrial calcium uniporter (MCU) is an important influx channel of mitochondrial Ca^2+^ homeostasis [24, 25] and Na^+^/Ca^2+^ exchanger (NCLX) is a major Na^+^/Ca^2+^ exchanger located in the inner membrane of mitochondria to efflux calcium [26, 27]. NCLX is essential in maintaining homeostasis, metabolic rate, and cell viability of cardiomyocytes [28]. For example, NCLX overexpression in PINK1 deficient cells protected neuronal cells from death in Parkinson’s disease [29]. Furthermore, PYK2 is a nonreceptor protein kinase activated by calcium and stimulates downstream kinases leading to proliferation [30]. Although cytosolic calcium signaling regulates cytotoxicity in chemotherapy [31] and cancer cells take advantage of cytosolic calcium increase to activate pathways involved in metastasis, proliferation, and stem cell enrichment [32], unfortunately, a role of NCLX in cancer chemotherapy is yet to be established.

In the present study, we explored the detailed mechanism of retrograde signaling by mitochondrial Lon on mtDNA damage upon cisplatin treatment. First, we showed that cisplatin caused mtDNA damage and upregulated the expression of Lon. Then we found that exogenous Lon upregulation leads to the resistance to and knockdown of Lon sensitizes the cells to cisplatin treatment. Moreover, the resistance by Lon is involved in the interaction with NCLX that increases the release of calcium from mitochondria to cytosol. This increased cytosolic calcium triggered calcium-dependent PYK2-SRC-STAT3 pathway to induce downstream expression of Bcl-2 and IL-6 that evade cell death and increase cell survival.

## Materials and methods

### Cell culture and cell treatment

OEC-M1, HSC-3, TW2.6 cells were cultured in a medium containing Dulbecco’s modified Eagle’s essential medium (DMEM) (GIBCO, New York, NY, USA), supplemented with 10% fetal bovine serum (FBS qualified; Invitrogen) and penicillin/streptomycin (50 U/ml, Sigma, St. Louis, MO, USA). Lon-shRNA: 5′-GAAAGUUCGUCUVGCCCAGCC-3′ (sh-1) and 5′-AGGAGCAGCUAAAGAUCAUCA-3′ (sh-2) [4]. Stable cell lines expressing Lon or Lon-shRNA were generated by retroviral infection using pMKO vector using the protocol from our previous work [4, 9]. Briefly, the plasmids, Lon-Sh1/2-pMKO-puro for stable knockdown cells, along with packaging plasmid gag-pol and envelope plasmid VSVG, were transfected into 293 T cells by Lipofectamine™ 2000 (Invitrogen, Carlsbad, CA, USA). Retroviral supernatant was collected at 48 h post-transfection and used to infect the target cells for 48 h. Puromycin (Sigma-Aldrich) was used to select the successfully infected cells at a final concentration of 2 μg/ml and the survived cells were collected to check the expression of human Lon by western blotting. Cultured cells were treated with hydrogen peroxide (200 μM H_2_O_2_, Sigma-Aldrich) for 4 h at 37 °C.

### Reagents and antibodies

Antibodies to human Lon were produced as described previously [33]. The antibodies used in this study were purchased as indicated: antibodies to PYK2(#3292), p-PYK2 (Tyr 402) (#3291), Src, p-Src (Tyr 416) (#2101 s), STAT3 (#GTX104616), p-STAT3 (Tyr 705) (#9131)were purchased from Cell Signaling Technology (Beverly, MA, USA) and GAPDH from Genetex (Hsinchu, Taiwan); NCLX from Sigma Aldrich (SAB2102181); GST antibody from Abnova (#MAB0042-M02); 8-OHdG antibody (E-8) was purchased from Santa Cruz Biotech Inc. (Dallas, TX, USA). Myc-Lon, shLon plasmids were produced as mentioned previously [9], NCLX plasmid was gifted by Dr. I. Sekler, Ben-Gurion University, Department of Physiology and Cell Biology, Israel.

### MTS assay

Cells were transiently transfected with vector, Lon, shLon plasmids for 48 h. After 48 h, transiently transfected cells were seeded cells into 96 well plate (otherwise stable cells directly transferred to 96 well plates) and cells were treated with cisplatin drug at indicated concentrations for 48 h or 24 h then treated with CGP37157 for other 24 h. MTS reagent was added according to the manufacturer’s protocol (Promega) for 90 mins and reading was done using Elisa reader at 490 nm. Cell viability was calculated and IC 50 was measured using Graph Pad Prism 5.0 version.

### Western blotting

Cells were scrapped with lysis buffer containing protease inhibitors prepared in-situ and kept in ice for 30 min and then centrifuged upon 13,000 rpm for 30 min to collect cell lysates. Lysates with sample buffer were heated to 100 °C and used for western blots. Briefly, samples were run on SDS- PAGE and transferred to PVDF membranes under 400 mA, 100 V, and blocked with 3% BSA in TBST for 1 hr. Washed thrice with TBST buffer and probed with primary antibody for overnight and then washed again and probed with secondary antibody for 1 h under room temperature and blots were developed using the chemiluminescent method.

### Transfection

Cells were seeded into plates and the next day transfection was done according to manufacturer’s protocol (Omics Bio). Briefly, maestrofectin reagent was added to serum-free medium and after 5 min plasmid was added and let the complex mixture to form for 15 min. These mixtures were added to cells and after 6 h, the medium was replaced with fresh medium and incubated for 48 h.

### Immunofluorescence

Cells were seeded into 6 well plate with coverslips. Next day, cells were rinsed with PBS and permeabilized with 4 % paraformaldehyde for 25 min at room temperature followed by fixation with ice-cold methanol. Then cells were blocked with 3% BSA and subjected to immunofluorescence staining with FITC 8-OHDG (1:50) from Santacruz (#sc-393871), TFAM (1:50) from Cell Signaling (#D5C8), or anti-Cisplatin modified DNA antibody from abcam (#ab103261) overnight at 4 ^o^C. Cells were then washed three times with PBS for 10 min each, and incubated with Alexa 594 anti-rabbit secondary antibody (1:350) (Invitrogen) at room temperature for 1 h. Then coverslips were mounted on glass slides using antifade DAPI mounting reagent. For double immunofluorescence staining, cells were incubated with Lon (1:50) antibodies overnight at 4 ^o^C temperature. The cells were washed with PBS, and incubated with Dylight-405 labeled anti-rabbit (1:150) at room temperature for 1 hr. Cells were then blocked with BSA for 1 h followed by staining with NCLX (1:50) antibody from Proteintech (#21430-1-AP) overnight at 4 ^o^C. Then cells were washed thrice with PBS and treated with Alexa 488- labeled anti-rabbit (1:350) secondary antibody (Genetex) at room temperature for 1 h. Later coverslips were mounted using Prolong Gold antifade mounting reagent (Invitrogen*)*. The cells were examined by confocal microscopy (Leica TCS SP5) with 63X objective.

### Calcium assay

For cytosol calcium measurement, cells were first transfected with Vector, Lon, shLon, or NCLX or treated with CGP37157 (10 µM), and after 6 h medium was replaced and treated with or without cisplatin (5 µM) for 48 h. After incubation, cells were washed with HBSS with Ca^2+^ and incubated with Fura-2 AM (1 µM) (molecular probes) for 30 mins at room temp. Then washed thrice with HBSS buffer and imaged under Leica microscope. Cells were imaged under 20X lens and captured using CCD camera every 0.5 s. Excitation was done at 340 and 380 nm alternatively and emission at 510 nm. Basal calcium levels were measured in presence of buffer and then agonists like ionomycin or ATP were added into buffers manually to further measure calcium. Analysis was done using LASX software and data was expressed as the ratio of 380/340 subtracted from background ROI. For mitochondrial calcium measurement, a lentiviral plasmid mt-lar-GECO [34] was purchased from Lumistar (Taipei, Taiwan) and a ratiometric pericam plasmid (a gift from Bjorn stork Add gene #87381) were used. Cells were seeded in 96-well plates and transducted with mt-lar-GECO for 24 h and then the medium was replaced and transfected with vector or NCLX or Lon plasmids for 48 h. After incubation, cells were washed with HBSS medium containing Ca^2+^ and assayed under Leica live imaging microscope. Plates were inserted into the chamber and solutions were changed manually. Cells were imaged under 40 × 1.5 numerical aperture and a CCD camera was used to capture the images every 2 sec. (Ca^2+^) _mito_ were measured by mt-lar-GECO with excitation 560/40 nm and emission 645/75 nm. First basal levels were measured in presence of HBSS buffer for 60 sec and later Cisplatin or ATP agonist was added and made further readings. Analysis was done using LASX software by choosing ROI. (Ca^2+^) _mito_ changes were quantified as (F-F0)/F0 where F is fluorescence intensity at each time point and F0 is the average fluorescence intensity of basal calcium. Cells were seeded in 6-well plates and transfected with ratiometric pericam along with vector or Lon plasmids for 48 h. After incubation, cells were washed with HBSS medium containing Ca^2+^ and assayed under Leica live imaging microscope. (Ca^2+^)_mito_ were measured by ratiometric pericam with alternative excitation of 380 and 488 nm filters and emission of 510 nm. First basal levels were measured in presence of buffer and later cisplatin or ATP agonist was added and made further readings. Analysis was done using LASX software by choosing ROI. Data expressed as the ratio of 488/380 subtracted from background ROI.

### Mitochondrial ROS detection

Cells were cultivated in 6 cm plates and cisplatin was treated for 48 h. Cells were then trypsinized and treated with mitosox for 30 min. Washed with PBS and analyzed by flow cytometer using FL-2 laser.

### GST pull-down assay

Lon plasmid was transfected into OEC-M1 cells and 48 h later cell lysates were collected. Lysates were immunoprecipitated using Lon antibody and eluted using 0.1 M glycine pH 2.0. Lon eluates were added to Glutathione sepharose beads (GE Health care) previously incubated with 1 µg of GST (Gifted by Dr. Hsu JT, NHRI, Taiwan) or GST-NCLX (Abnova # H00080024-P01) recombinant proteins and end on end mixing was done for 4 h. Beads were then washed thrice with lysis buffer having cocktail inhibitors. Later sample buffer was added and heated followed by Western blot.

### In vivo tumor xenograft experiment using mouse model

BALB/C Nu mice were purchased from National Laboratory Animal Center (Taiwan). The OEC-M1 cell overexpressing Lon was rinsed twice with PBS and resuspended in PBS with Matrigel Matrix (Corning), with the cell number regulated to 1 × 10^6^ /ml. OEC-M1 cells overexpressing Lon were injected subcutaneously into BALB/C Nu mice. Each mouse was injected subcutaneously at one side. After 1 week, when the tumors had grown to 50–100 mm^3^, the tumor-bearing nude mice were randomly divided into four groups (*n* = 6 per group) as follows:

The mice bearing tumor were pre-treated with cisplatin via intraperitoneal injection (i.p.) at 10 and 12 days postinjection. Different combinations and time of treatment were used as indicated. The tumor size (above) and weight (below) were measured before every point or injection. Control, Cisplatin, CGP37157, or Cisplatin/CGP37157 were used to check tumorigenicity of Lon-overexpressing cells. Data represented are the mean of *n* = 6 mice. The error bars represent the standard deviation from six independent mice. Animals were randomized before experiments. Procedures involving mice and their care were in accordance with the guidelines of the Laboratory Animal Center of National Heath Research Institutes (Taiwan), which were developed in accordance with the Guide for the Care and Use of Laboratory Animals (National Academy of Sciences, USA, 1985).

### Patients and clinical sample

Tissue specimens of 6 patients with oral squamous cell carcinoma (OSCC) were used for immunohistochemistry (IHC) analysis based on the availability of archival human tissue blocks from diagnostic resection specimens in the Departments of Pathology at Mackay Memorial Hospital, Taipei, Taiwan with approval from the Institutional Review Board (IRB numbers 17MMHIS085). All experiments were performed in accordance with relevant guidelines and regulations.

### Immunohistochemistry

Paraffin-embedded tissues were deparaffinized and subjected to antigen retrieval with citrate buffer pH 6.0 and treated with 3% hydrogen peroxide to block endogenous peroxidase activity. Tissues were blocked with serum and single staining was done by incubating primary antibody Lon (LSBio #LS-C752623) or NCLX (Proteintech #21430-1-AP) at 4 ^o^C overnight. Slides were stained with biotin-conjugated antibody, streptavidin peroxidase (VECTA STAIN Elite ABC staining kit, VECTOR laboratories, USA) followed by substrate addition (VECTOR NovaRED substrate kit, peroxidase VECTOR laboratories, USA) to observe immnoreactivity. Slides were scanned using PANNORAMIC Digital slide scanner.

### Statistical analysis

Significance of differences between means of samples was established using one-way ANOVA with Tukey’s post-test analysis or two-way ANOVA with Bonferroni post-test analysis. ***P < 0.0001, **P < 0.01, *P < 0.05.

## Results

### Cisplatin-induced Lon contributes to the cytotoxicity resistance of cisplatin in OSCC cells

**Fig. 1:**
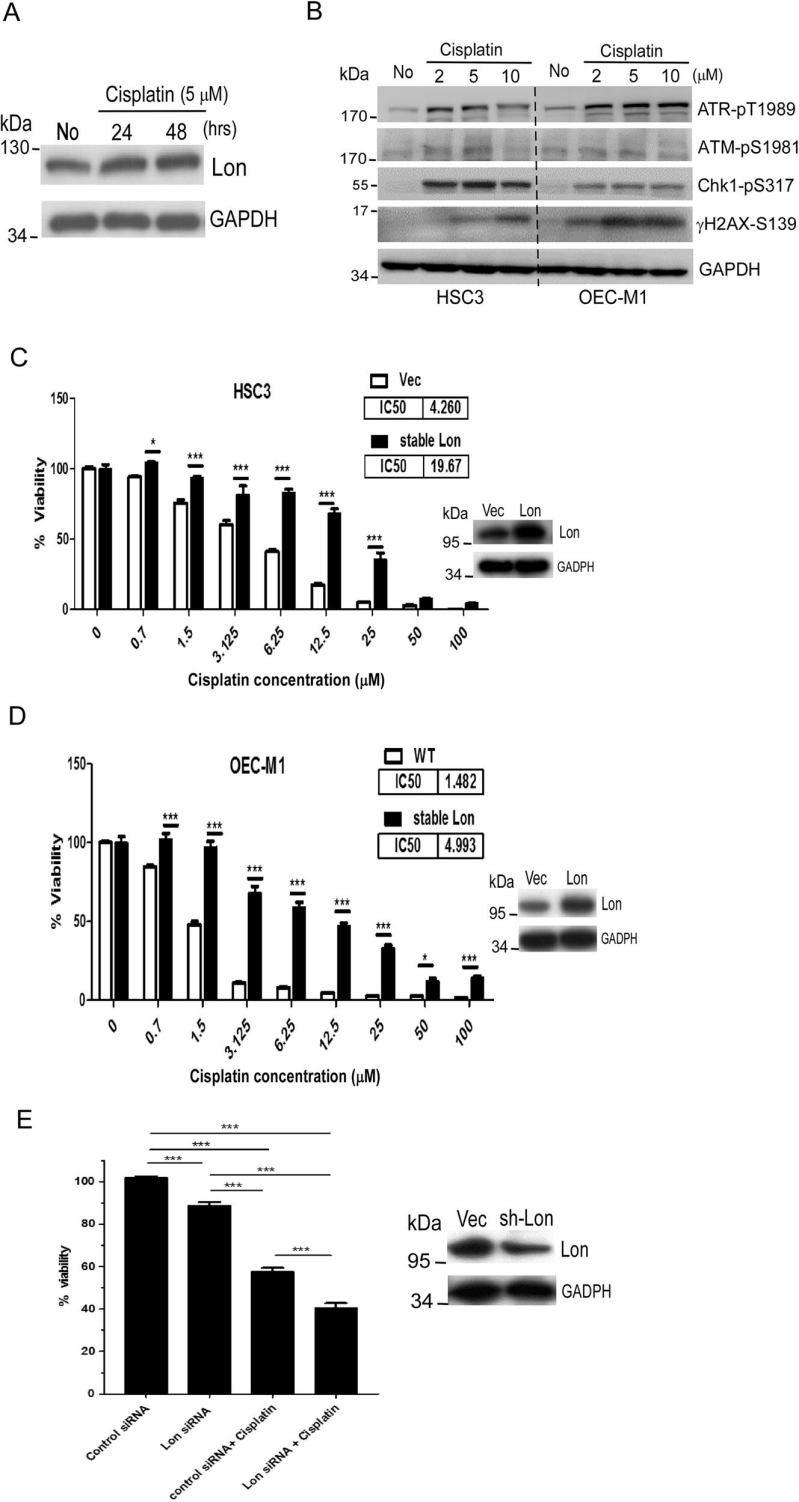
Lon overexpression increases the resistance of cisplatin on oral cancer cells. **A** and **B**. Cisplatin treatment induces Lon expression and causes DNA damage **A** OEC-M1 cells were treated with cisplatin at indicated time points and cell lysate was collected. Western blot was performed using indicated antibodies. **B** OEC-M1 and HSC3 cells were treated with cisplatin at indicated concentrations and Western blot was performed using indicated antibodies. C and D. Lon overexpression increases the viability of oral cancer cells upon cisplatin treatment. Cisplatin cytotoxicity was assayed in HSC3 **(C)** and OEC-M1 **(D)** cells overexpressing Lon or not following cisplatin treatment at different concentrations for 48 h. Cell cytotoxicity was performed using MTS assay. IC50 values were measured by using Log values using Graph Pad Prism 5.0 version software. **E**. Lon downregulation sensitize the cytotoxicity of cisplatin on oral cancer cells Lon expression was knocked down in OEC-M1 cells by using scramble shRNA or Lon-shRNA. The cells were treated with cisplatin at different concentrations for 48 h and cell cytotoxicity was measured using MTS assay. The experiments were done at least three times and ***p < 0.001 denotes significance.

We hypothesized that mitochondrial Lon upregulation leads to the resistance to cisplatin treatment in OSCC cells. Cisplatin forms adducts with DNA and induces DNA damage responses and apoptosis. In addition, cisplatin is accumulated in mitochondria and targets mitochondrial DNA (mtDNA) and proteins, which induces the production of mitochondrial ROS [19, 35, 36]. We indeed observed that Lon protein expression is elevated upon cisplatin treatment in OEC-M1 oral cancer cells in a time-dependent manner (Fig.1A). In addition, DNA adducts produced by cisplatin cause nuclear DNA damage response in a dose-dependent manner (Fig.1B). To ensure that cisplatin treatment induced the expression of Lon, we further analyzed an independent dataset examining the mitochondrial proteome of cisplatin-sensitive and resistant ovarian cancer cells [37]. We found that cisplatin-resistant cells showed increased Lon expression compared to sensitive cells by a fold change of 2.24 (Fig. S1A). To prove Lon has a protective role in cisplatin cytotoxicity, we used Lon-overexpressing cells to evaluate the effect of cisplatin cytotoxicity. We treated Lon-overexpressing cells with increased concentrations of cisplatin for 48 h compared to the control. Lon upregulation increased proliferation and increased IC50 of cisplatin in comparison to the control in HSC3, OEC-M1, and TW2.6 cells (Figs. 1C, D and S1B). Consistently, Lon knocking-down OEC-M1 cells with Lon-siRNA sensitized cell cytotoxicity toward cisplatin compared to control siRNA in OEC-M1 cells (Fig. 1E). We concluded that mitochondrial Lon overexpression leads to the resistance to cisplatin treatment in OSCC cells.

### Cisplatin treatment causes ROS-dependent DNA damage in the nucleus and mitochondria

**Fig. 2:**
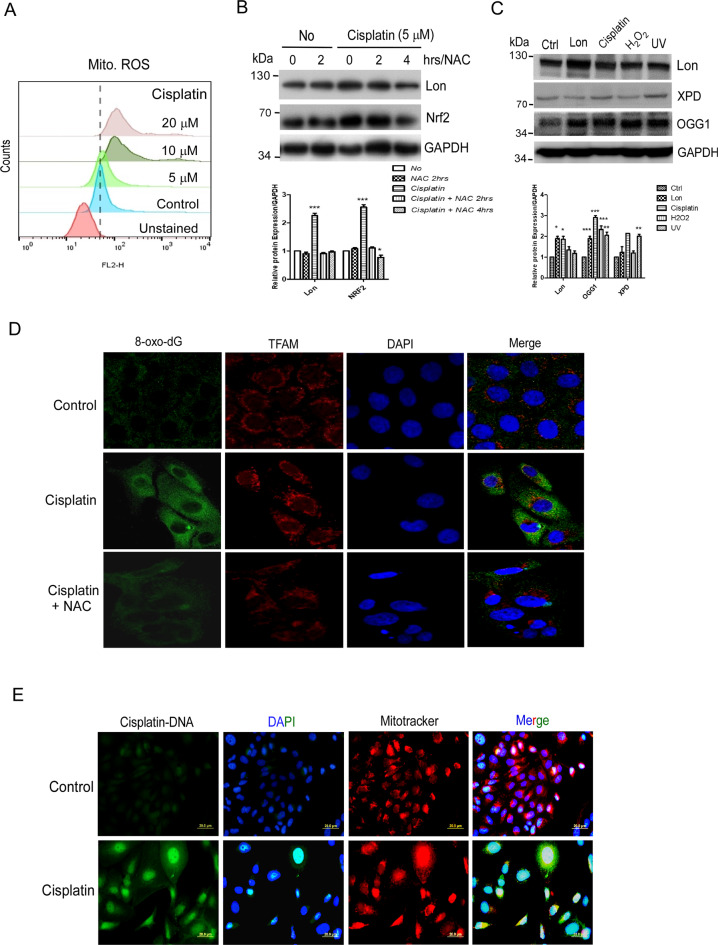
Cisplatin treatment causes ROS-dependent mitochondria DNA damage. **A** Cisplatin treatment triggers mitochondrial ROS production. OEC-M1 cells were treated with different concentrations of cisplatin and mitochondrial ROS levels were detected using Mitosox dye. **B** Cisplatin treatment induces Lon expression in a ROS-dependent manner. OEC-M1 cells were treated with or without NAC (5 mM), a ROS scavenger, for indicated time points followed by with or without treatment with cisplatin for 48 h. Cell lysates were collected and Western blot was performed by using indicated antibodies. **C** Cisplatin and Lon overexpression causes oxidative DNA damage OEC-M1 cells were transfected with vector or Lon plasmid or treated with cisplatin. Western blot was performed using indicated antibodies. H_2_O_2_ and UV treatment acts as positive controls. Western blot was performed using indicated antibodies. **D** and **E** Cisplatin binds to mitochondrial DNA (mtDNA) and causes oxidative damage in mtDNA. **D** OEC-M1 cells were treated with or without NAC 5 mM for 4 h followed by incubation with or without cisplatin for 48 h and immunofluorescence staining was performed using 8-oxo-dG (green), TFAM (red) and merged images with DAPI (blue). **E** OEC-M1 cells were treated with or without cisplatin (5 µM) for 48 h and immunofluorescence staining was performed using anti-Cisplatin-DNA adducts antibody (green), Mitotracker (red) and merged images with DAPI (blue). Scale bar, 20 μm.

We next asked how mitochondrial Lon overexpression leads to the resistance to cisplatin treatment. We found that cisplatin treatment on OEC-M1 cells increased mitochondrial ROS in a dose-dependent manner compared to the control (Fig. 2A), and cisplatin-induced Lon expression could be reduced by the treatment of NAC (N-Acetyl Cysteine) (Fig. 2B). We also observed that Lon protein expression is elevated upon cisplatin, UV treatment, and oxidative stress in oral cancer cells (Fig. 2C), which is consistent with our findings that oxidative stress induces Lon upregulation and Lon induces mitochondrial ROS production [9]. To investigate cisplatin treatment leads to mtDNA oxidative damage, we first checked the accumulation of XPD, a nucleotide excision repair (NER) helicase, after Lon overexpression or the treatment of cisplatin, UV, or oxidative stress. We found that XPD protein expression was increased and accumulated upon UV treatment but not cisplatin treatment, hydrogen peroxide (H_2_O_2_) treatment, or Lon overexpression in oral cancer cells (Fig. 2C), suggesting that cisplatin and Lon overexpression causes mtDNA oxidative damage more than the adduct damage. To prove cisplatin treatment leads to mtDNA oxidative damage, we checked the mitochondrial accumulation of 8-oxodG glycosylase 1 (OGG1), a base excision repair (BER) enzyme. We found that OGG1 protein expression is increased upon cisplatin, H2O2 treatment, or Lon overexpression in oral cancer cells (Fig. 2C), suggesting that cisplatin/Lon causes oxidative damage in mtDNA. We confirmed that cisplatin treatment leads to mtDNA oxidative damage by observing the accumulation of 8-oxo-2’-dihydroguanine (8-oxo-dG) in mtDNA (Fig. 2D), which is a biomarker of oxidative damaged DNA and repaired by OGG1 [38] and which can be inhibited by the treatment of NAC (Fig. 2D). We used TFAM as a mtDNA biomarker. To further confirm cisplatin directly targets mtDNA and causes oxidative damage, we checked the mitochondrial accumulation of cisplatin-DNA adducts by using anticisplatin-modified DNA antibody. We confirmed that mtDNA damage is caused by direct binding with cisplatin (Fig. 2E), suggesting that cisplatin targets mtDNA and causes oxidative damage in mtDNA. Together, these results show that cisplatin-induced oxidative stress causes nuclear and mitochondrial DNA damage and increases Lon expression and ROS in oral cancer cells.

### Lon promotes cell survival under cisplatin treatment by activating the PYK2-SRC-STAT3-IL-6 pathway

**Fig. 3:**
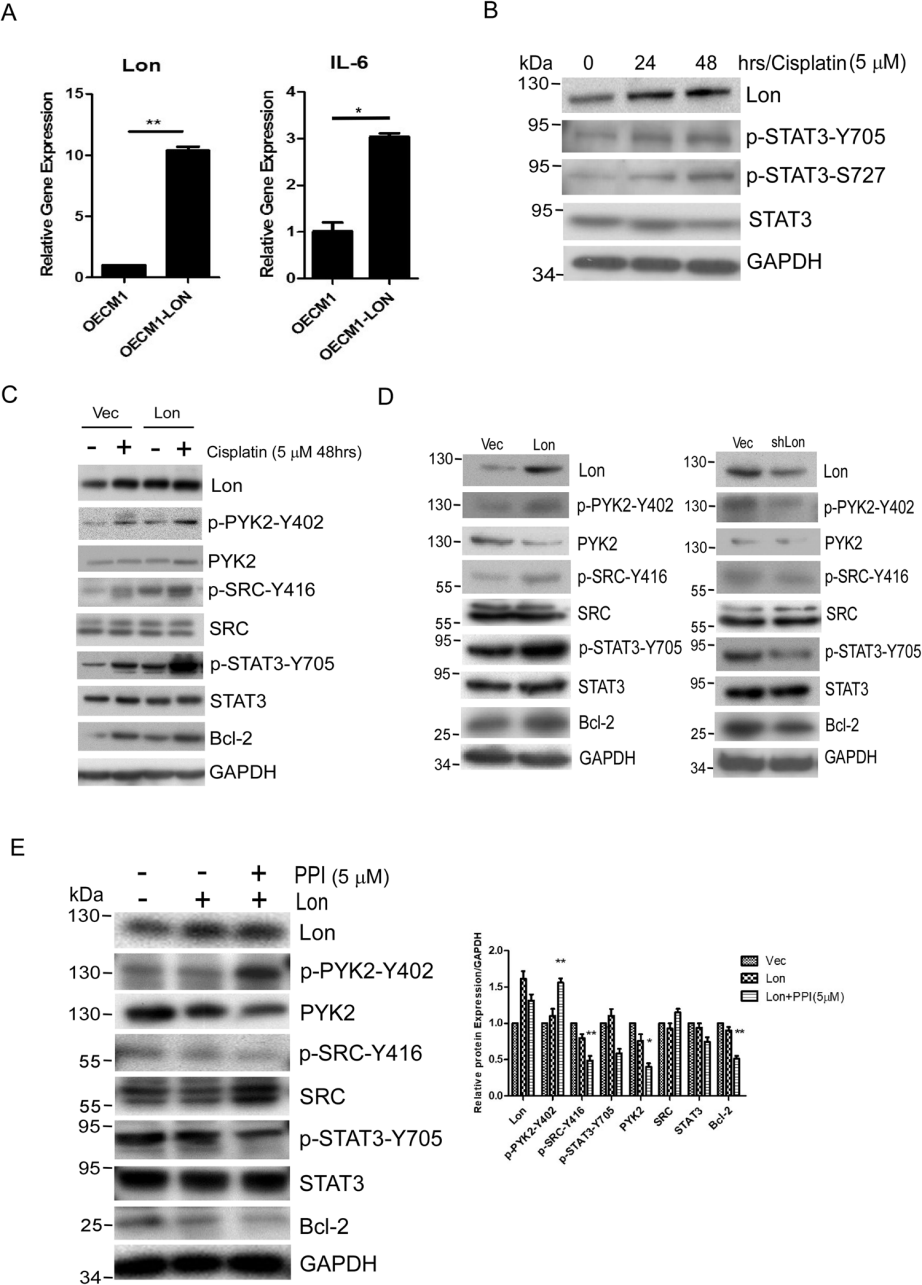
Mitochondrial Lon activates Ca^2+^-PYK2-SRC-STAT3-IL-6 pathway under cisplatin treatment. **A** Lon overexpression promotes IL-6 expression in oral cancer cells. OEC-M1 cells were transfected with or without Lon plasmid for 48 h. IL-6 mRNA expression was detected by qPCR. Lon expression acts as a positive control (left panel). **B** Cisplatin treatment activates Lon expression and STAT3 pathway OEC-M1 cells were treated with cisplatin (5 µM) for different time points and Western blot was performed using indicated antibodies. **C** OEC-M1 cells transfected with or without Lon plasmid were treated with or without cisplatin (5 µM) for 48 h. Western blot was performed using indicated antibodies. **D** OEC-M1 cells were transfected with Lon or Lon-shRNA plasmid and Western blot was performed using indicated antibodies. **E**. OEC-M1 cells transfected with or without Lon plasmid were treated with PPI (SRC inhibitor, 5 µM) for 4 h. Western blot was performed using indicated antibodies.

We tried to find the mechanism of mitochondrial Lon overexpression leads to the resistance to cisplatin treatment. Our previous study showed that mitochondrial Lon upregulation increases IL-6 expression and promotes cancer progression and metastasis [12]. It is well known that IL-6 expression is mediated by STAT3, hinting that Lon regulates STAT3 to activate IL-6 increase for cell survival upon cisplatin treatment [32]. We then confirmed that mitochondrial Lon overexpression increases IL-6 expression (Fig. 3A). Interestingly, cisplatin treatment induced Lon expression and the activation of p-STAT3 (Fig. 3B). These results suggest that cisplatin-induced Lon that contributes to cell survival may be involved in inflammatory STAT3-IL-6 signaling. Since PYK2 is a calcium-activated tyrosine kinase and enriches cancer stem cells by activating SRC-STAT3 signaling upon carboplatin treatment [32, 39], we decided to check whether cisplatin treatment-induced Lon activates the calcium-related PYK2-SRC-STAT3 pathway. The results showed that cisplatin treatment and Lon overexpression triggers the activation of PYK2-SRC-STAT3 pathway (Fig. 3C). Moreover, cisplatin-induced STAT3 stimulated its target gene Bcl-2 significantly in Lon-overexpressed cells, which is resistant to apoptosis (Fig. 3C). Similarly, in physiological condition, Lon overexpression activated and downregulation decreased PYK2-SRC-STAT3 axis in OEC-M1 cells (Fig. 3D), indicating Lon as an upstream activator of the pathway. Further pharmacological inhibition of SRC by PPI abrogated STAT3 phosphorylation and Bcl-2 but not upstream proteins p-PYK2 and Lon (Fig. 3E). These results indicate that cisplatin-induced Lon activates the cell survival pathway PYK2-SRC-STAT3-IL-6/Bcl-2.

### Lon increases cytosol calcium via mitochondrial Ca^2+^/Na^+^ exchanger NCLX

**Fig. 4:**
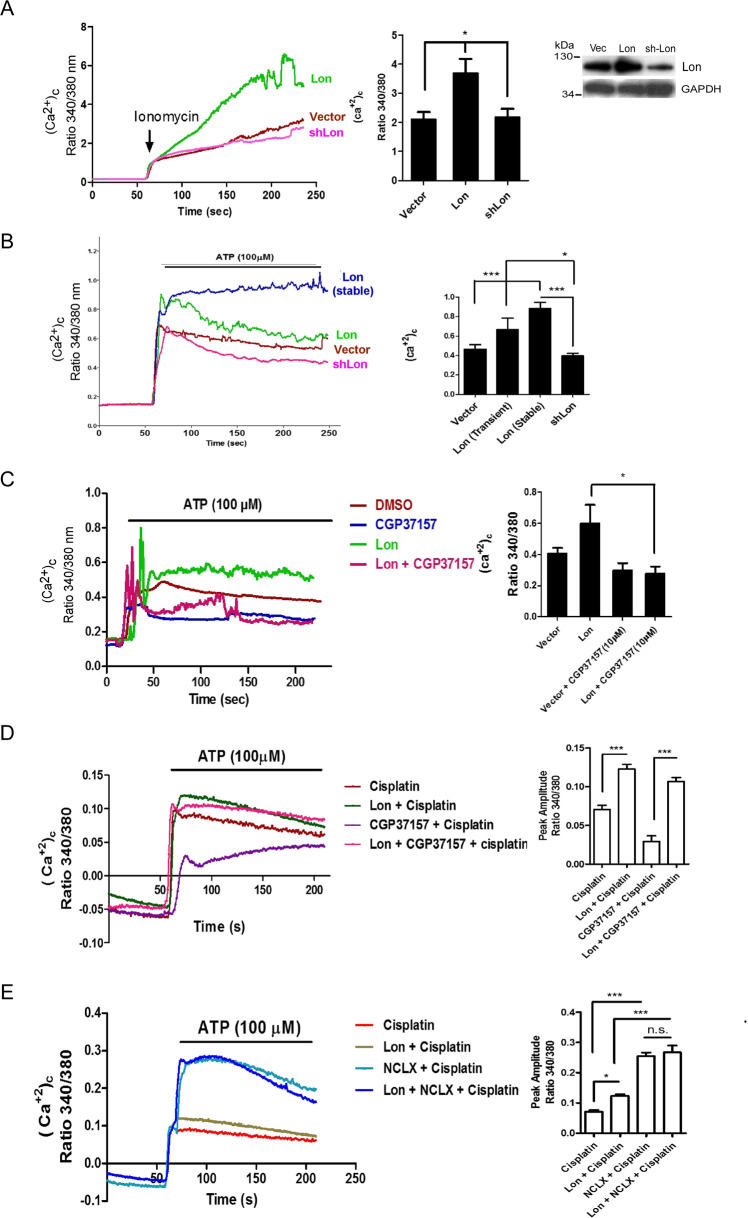
Mitochondrial Lon increases cytosolic Ca^2+^ level through NCLX activity. **A** and **B** Lon overexpression increases cytosolic Ca^2+^ level in oral cancer cells. OEC-M1 cells transfected with Lon or Lon-shRNA plasmids were stimulated with ionomycin (*N* = 20) and cytosolic calcium was measured using Fura-2 AM dye (1 μM). Representative plot of the intensity of fluorescent images was shown. The error bars shown in the right panel represent the standard deviation from three independent experiments. *p < 0.05 **(A)**. OEC-M1 cells transfected with Lon or Lon-shRNA plasmids or stably Lon-overexpressing OEC-M1 cells (Lon stable) were stimulated with ATP and cytosolic calcium were measured using Fura-2 AM dye (1 μM) (*N* = 20). Representative plot of the intensity of fluorescent images was shown. The error bars shown in the right panel represent the standard deviation from three independent experiments. *p < 0.05; ***p < 0.001 **(B)**. **C** OEC-M1 cells transfected with or without Lon plasmids were treated with CGP37157 (NCLX inhibitor, 10 μM) or not and cytosolic calcium was stimulated with ATP and measured using Fura-2 AM dye (1 μM) (*N* = 15). Representative plot of the intensity of fluorescent images was shown. The error bars shown in the right panel represent the standard deviation from three independent experiments. *p < 0.05. **D**. OEC-M1 cells were treated with cisplatin (5 mM) in vector or Lon transfected cells followed by treatment with or without CGP37157(10 μM). Cytosolic calcium was stimulated with ATP and measured using Fura-2 AM dye (1 μM) (*N* = 15). Representative plot of the intensity of fluorescent images was shown. The bar graph on the right represent the peak amplitude intensity and the error bars shown represent the standard deviation from three independent experiments. ***p < 0.001. **E** OEC-M1 cells were co-transfected with Lon and/or NCLX plasmids along with vector and treated with or without cisplatin (5 mM) were stimulated with ATP and cytosolic calcium was measured using Fura-2 AM dye (1 μM) (*N* = 15). Representative plot of the intensity of fluorescent images was shown. The bar graph on the right represent the peak amplitude intensity and the error bars shown represent the standard deviation from three independent experiments. ***p < 0.001, *p < 0.05, n.s. non-significant. Lon and vector treated with cisplatin were used as positive controls.

Since the pathway may be involved in calcium-dependent PYK2 activation, we investigated whether cisplatin-induced Lon overexpression triggers an increase in cytosolic calcium. We monitored cytosol calcium levels by using Fura-2 AM dye and using ionomycin and ATP as agonists. We found that cytosolic calcium levels in Lon-overexpressing cells were significantly increased, and conversely the levels were decreased upon Lon downregulation (Figs. 4A, B and S2A). These results convey that Lon increases the level of calcium intracellularly to activate PYK2-SRC-STAT3 signaling involved in cell survival. We next asked how mitochondrial Lon upregulates cytosol calcium level. Since the expression of NCLX, a mitochondrial Ca^2+^/Na^+^ exchanger, was also increased in cisplatin-resistant ovarian cancer cells (Fig. S1A) [37], we hypothesized that Lon increases cytosol calcium level through upregulating the activity of NCLX, which decreases mitochondrial calcium level simultaneously. To check this, we first used NCLX inhibitor CGP37157 at different concentrations to treat OEC-M1 cells and found that cytosol calcium level was decreased in a dose-dependent manner (Fig. S2B). We then examined the role of NCLX in Lon overexpression-induced increase in cytosolic calcium. Consistently, cytosolic calcium level was increased when Lon was overexpressed and NCLX inhibitor treatment significantly abolished Lon-induced increase in cytosolic calcium (Figs. 4C and S2C), suggesting that cytosol calcium increase by Lon is involved in the activity of NCLX exchanger. Next we examined whether cisplatin-induced Lon overexpression triggers an increase in cytosolic calcium through NCLX exchanger. We found that cytosolic calcium level was increased when Lon was overexpressed and/or cisplatin treatment; however, NCLX inhibitor treatment significantly abolished the increase in cytosolic calcium (Fig. 4D). This result was confirmed by that overexpression of NCLX further increased cytosolic calcium levels compared to the Lon overexpression and/or cisplatin treatment (Fig. 4E).

**Fig. 5:**
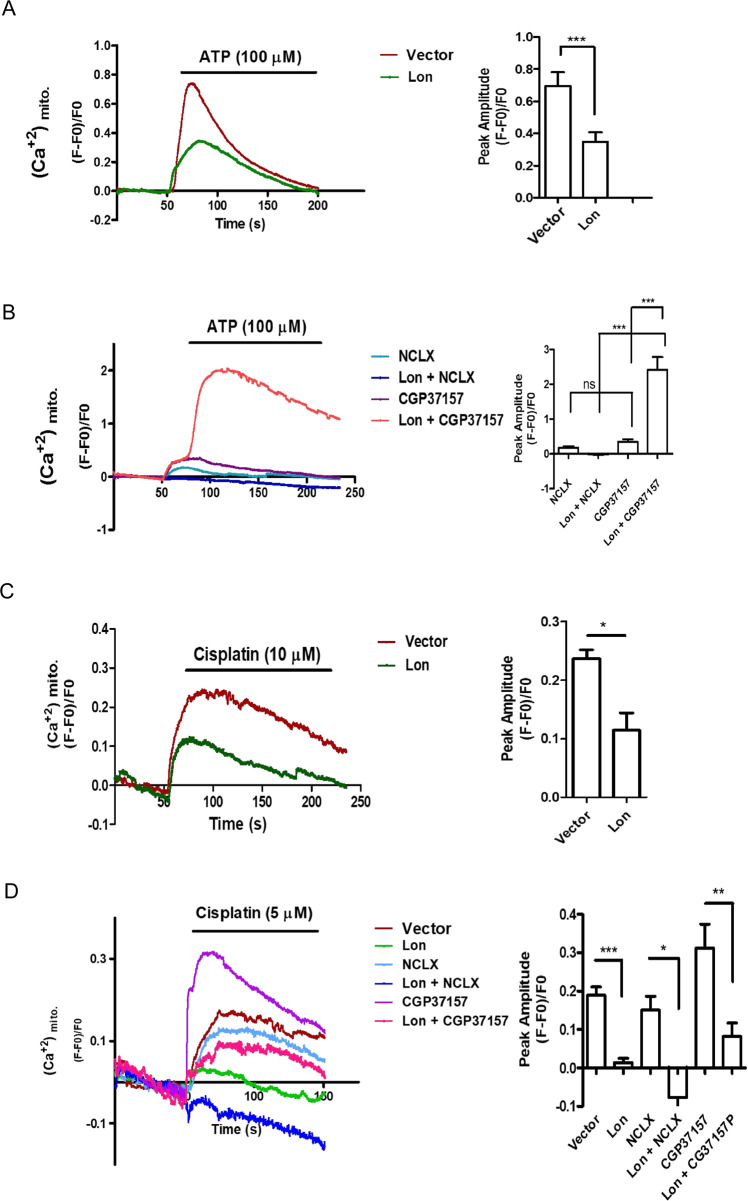
Mitochondrial Lon decreases mitochondria Ca^2+^ level through NCLX activity. **A** OEC-M1 cells transfected with Lon or vector plasmids were stimulated with ATP (*N* = 30) and mitochondrial calcium was measured using mt-lar-GECO. Representative plot of the intensity of fluorescent images was shown. The bar graph on the right represent the peak amplitude intensity and the error bars shown represent the standard deviation from three independent experiments. ***p < 0.001. **B** OEC-M1 cells transfected with vector or Lon plasmids were either co-transfected with NCLX or treated with CGP37157 (10 μM). Mitochondria calcium was stimulated with ATP (*N* = 30) and measured using mt-lar-GECO. Representative plot of the intensity of fluorescent images was shown. The bar graph on the right represent the peak amplitude intensity and the error bars shown represent the standard deviation from three independent experiments. ***p < 0.001, n.s. nonsignificant. **C** OEC-M1 cells transfected with vector or Lon plasmids were stimulated with cisplatin (10 μM) and mitochondria calcium was measured using mt-lar-GECO. Representative plot of the intensity of fluorescent images was shown. The bar graph on the right represent the peak amplitude intensity and the error bars shown represent the standard deviation from three independent experiments. ***p < 0.001. **D** OEC-M1 cells expressed with vector or Lon were co-transfected with or without NCLX plasmids or treated with or without CGP37157 were stimulated with cisplatin and mitochondrial calcium was measured using mt-lar-GECO. A representative plot of the intensity of fluorescent images was shown. The error bars shown in the right panel represent the standard deviation from three independent experiments. *p < 0.05; **p < 0.01, ***p < 0.001. Lon and vector cells were used as positive controls.

Next we tested the role of Lon in the regulation of mitochondrial calcium levels under cisplatin treatment. The mitochondria calcium levels were detected by using mt-lar-GECO or ratiometric pericam fluorescent sensor [40, 41]. Mitochondrial calcium levels indeed were decreased in Lon-overexpressing OEC-M1 cells (Figs. 5A and S3A) and further decreased in the cells co-overexpressed with NCLX (Figs. 5B and S3A) or increased in the cells co-treated with NCLX inhibitor CGP37157 (Fig. 5B). We then tested whether cisplatin treatment regulates the mitochondrial calcium. We found that mitochondrial calcium levels were increased in the cisplatin-treated cells (Fig. 5C); overexpression of Lon and/or NCLX further reduced mitochondrial calcium levels under cisplatin treatment compared to the control (Figs. 5D and S3B). However, the treatment of NCLX inhibitor CGP37157 increased mitochondrial calcium levels in Lon-overexpressing OEC-M1 cells; overexpression of NCLX decreased mitochondrial calcium levels in Lon-overexpressing OEC-M1 cells (Fig. 5D). Taken together, these results indicate that mitochondrial Lon increases cytosol calcium levels upon cisplatin treatment through the activity of NCLX, a mitochondrial Ca^2+^/Na^+^ exchanger.

### Mitochondrial Lon interacts and activates NCLX

**Fig. 6:**
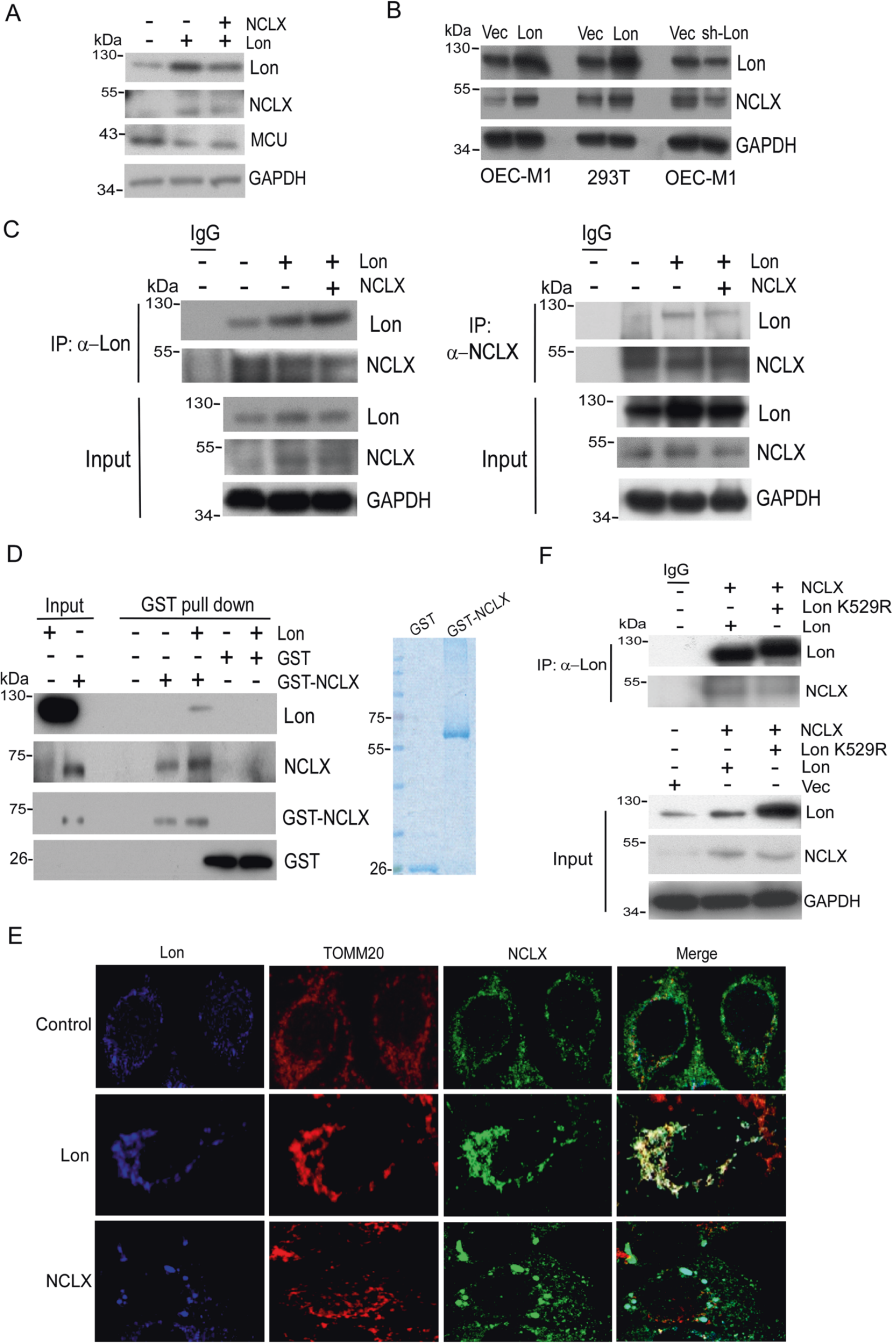
Mitochondrial Lon interacts with and stabilizes NCLX. **A** OEC-M1 cells were transfected with Lon and/or NCLX plasmids and Western blot was performed using indicated antibodies. **B** OEC-M1 and 293 T cells were transfected with Lon or Lon-shRNA plasmids and Western blot was performed using indicated antibodies. **C** Mitochondrial Lon interacts with NCLX shown by co-immunoprecipitation. OECM-1 cells were transiently transfected with the plasmids encoding Lon and/or NCLX followed by co-immunoprecipitation with anti-Lon and anti-NCLX, respectively. Whole cell lysates from OECM-1 cells transfected with the plasmids encoding Lon and/or NCLX were immunoprecipitated with anti-Lon or anti-NCLX antibodies. The immunoprecipitation complex was analyzed by Western blotting using the indicated antibodies. IP, immunoprecipitation. **D** Mitochondrial Lon interacts with NCLX shown by GST Pull-down assay. GST Pull-down assay was performed by GST-NCLX recombinant protein (1 µg) and antibody-purified Lon by using glutathione sepharose beads. GST (1 µg) was used as a negative control. GST-NCLX and GST recombinant protein (1 µg) were shown in the commassive stain gel (right panel). Western blots were performed using indicated antibodies. **E** The chaperone activity of mitochondrial Lon is required for the interaction with NCLX. OEC-M1 cells were cotransfected with Lon or Lon K529R and NCLX plasmids followed by co-immunoprecipitation with anti-Lon. Western blot was done using indicated antibodies to check the immunoprecipitation complex. IP, immunoprecipitation. **F** Immunofluorescence staining was performed on OEC-M1 cells transiently transfected with vector or Lon or NCLX plasmids using NCLX (green), TOMM20 (red) mitochondria marker and Lon (blue) and merged images were shown.

**Fig. 7:**
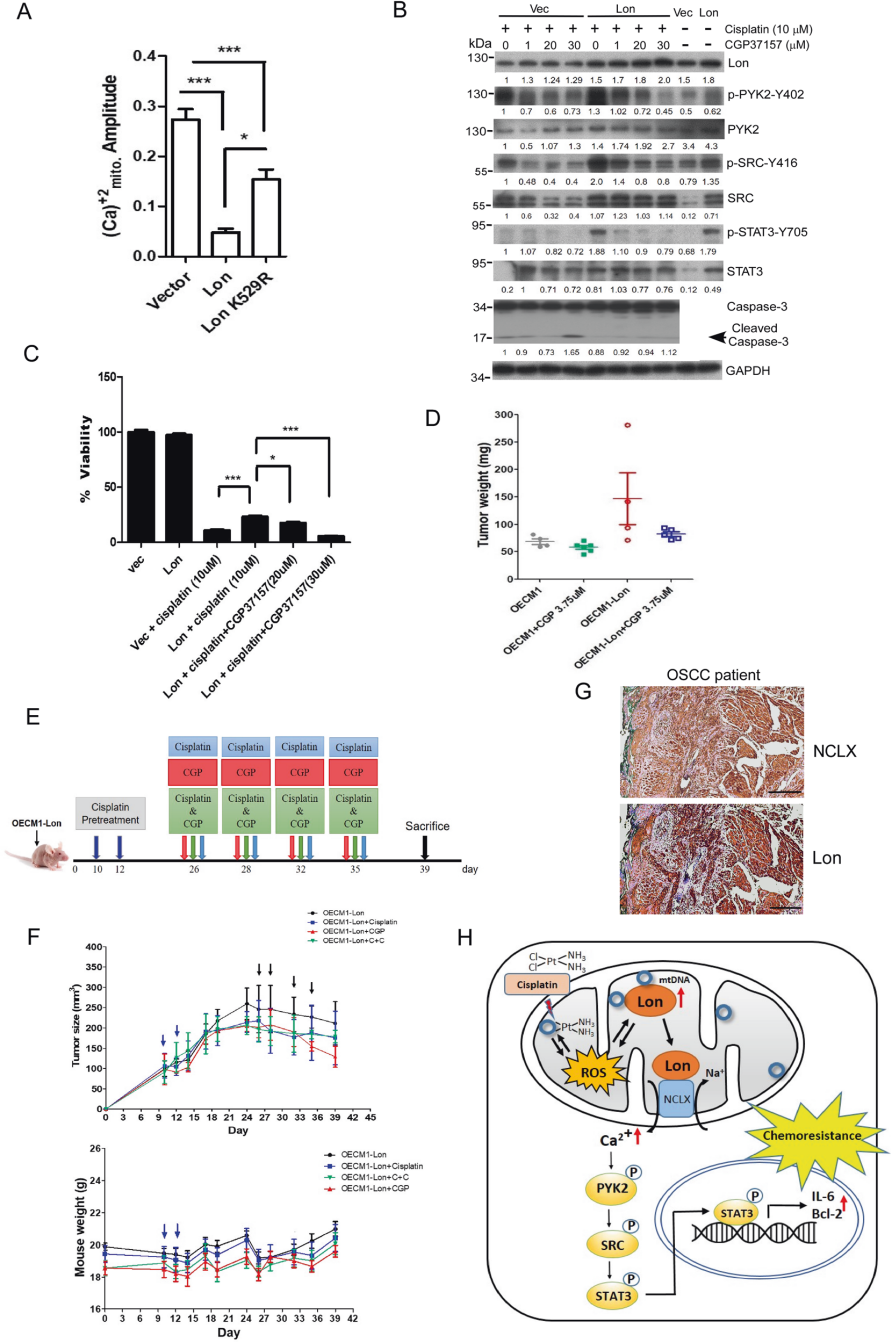
Mitochondria Lon-induced cisplatin resistance is mediated by mitochondrial Ca2 + -dependent signaling. **A**. Lon-induced mitochondrial Ca2 + efflux is dependent on the interaction with NCLX under cisplatin treatment OEC-M1 cells transfected with vector, Lon, or Lon K529R plasmids were stimulated with cisplatin (10 μM) and mitochondria calcium was measured using mt-lar-GECO. Representative plot of the intensity of fluorescent images was shown. The bar graph on the right represent the peak amplitude intensity and the error bars shown represent the standard deviation from three independent experiments. ***p < 0.001. **B** OEC-M1 cells transfected with or without Lon plasmid were treated with cisplatin (5 µM) and/or CGP37157 at indicated concentrations for 48 h. Western blot was performed using indicated antibodies. **C**. OEC-M1 cells transfected with vector or Lon plasmids treated with cisplatin and/or CGP37157 and cell cytotoxicity was measured by using MTS assay. Data were shown as a representative of three separate experiments. ***p < 0.001, *p < 0.05. **D** CGP37157 treatment inhibits Lon-induced tumorigenesis in vivo. OEC-M1 cells overexpressing Lon or not were injected subcutaneously into BALB/C Nu mice. The mice bearing tumor were treated with the dose of CGP37157 as indicated via intraperitoneal injection (i.p.) 7 days postinoculation. The tumor weight was measured at the endpoint. Each dot represents one mouse. **E** and **F.** The effect of CGP37157 on the drug resistance after prolonged cisplatin treatment under Lon overexpression in vivo. (**E**) Design of experimental protocol. (**F**) CGP37157 treatment overcomes the Lon-induced drug resistance under cisplatin treatment in vivo*.* OEC-M1 cells overexpressing Lon were injected subcutaneously into BALB/C Nu mice. The mice bearing tumor were pretreated with cisplatin via intraperitoneal injection (i.p.) at 10 and 12 days postinoculation. Different combinations and time of treatment were used as indicated in **(E)**. The tumor size (above) and weight (below) were measured before every point or injection. Data represented are the mean of *n* = 6 mice. The error bars represent the standard deviation from six independent mice. **G.** Immunohistochemical analysis of NCLX and Lon expression in OSCC patients. Representative immunohistochemical staining of NCLX and Lon was performed using paraffin-embedded sections of OSCC. Microscopic magnification, 200×. Scale bar, 200 μm. **H.** The model depicts how Lon upregulation by cisplatin contributes to the resistance by regulating cytosolic Ca^2+^ level in cancer cells. Cisplatin treatment causes mitochondrial DNA (mtDNA) damages and induces mitochondrial oxidative stress. Cisplatin-induced ROS further induce Lon protein expression that is a mtDNA-binding protein. Mitochondrial Lon acts as a chaperone to bind and activate NCLX to release mitochondrial calcium (Ca^2+^) to the cytosol. Cytosolic Ca^2+^ thereby stimulates the PYK2-SRC-STAT3 signal pathway. Activated STAT3 translocates to nucleus to activate IL-6 and Bcl-2 expression that increases the survival of cancer cells leading to cisplatin resistance.

We next sought to investigate the relationship between Lon and NCLX. We first checked the expression of NCLX upon differential expression of Lon. We found that Lon overexpression increased the expression of NCLX in OEC-M1 cells (Fig. 6A). Consistently, Lon overexpression increased and Lon knock-down decreased the expression of NCLX in OEC-M1 and 293 T cells (Fig. 6B), suggesting that NCLX may be a client of chaperone protein Lon. In our previous work, we have shown that mitochondrial Lon acts as a chaperone protein that maintains the stability of its client proteins by interacting with them [4, 12, 42]. Thus, we suspected Lon interacts with NCLX to maintain its stability and activity. We then performed coimmunoprecipitation assay and found that NCLX was co-immunoprecipitated by using Lon antibody, and vice versa (Fig. 6C). Furthermore, the GST-tag pull-down assays confirmed a direct interaction between Lon and NCLX in vitro by using purified Lon and GST-NCLX proteins (Fig. 6D). Consistently, the immunofluorescence results showed that Lon overexpression increased the expression of NCLX in OEC-M1 cells (Fig. 6E). The interaction or colocalization between Lon and NCLX was increased in both Lon overexpression and NCLX overexpression group (Fig. 6E). We then tested the role of Lon chaperone activity in the interaction between Lon and NCLX and in the regulation of mitochondrial calcium levels. We first found that the interaction between Lon and NCLX was decreased in the ATPase mutant LonK529R group (Top panel, Fig. 6F) and the LonK529R overexpression decreased the expression of NCLX (Bottom panel, Fig. 6F). Consistently, overexpression of the LonK529R mutant increased mitochondrial calcium levels under cisplatin treatment compared to the Lon WT (Fig. 7A). These results indicated that mitochondrial Lon modulates cytosol/mitochondrial Ca^2+^ levels upon cisplatin treatment through interacting and increasing the activity of NCLX.

### Mitochondria Lon-induced cisplatin resistance is mediated by mitochondria Ca^2+^--dependent signaling

We next questioned whether Lon-induced cisplatin resistance is mediated by calcium release from mitochondria and Ca^2+^-dependent PYK2-SRC-STAT3 pathway. To corroborate the signaling pathway is calcium dependent, we used NCLX inhibitor CGP37157 to treat the Lon-overexpressing or control cells in presence of cisplatin. We observed that cisplatin treatment activated the PYK2-SRC-STAT3 signaling and the activation was significantly increased in Lon overexpressing cells compared to control cells. However, the activation of PYK2-SRC-STAT3 signaling was decreased by NCLX inhibitor treatment in a dose-dependent manner (Fig. 7B). In addition, the amount of cleaved caspase-3 was increased by NCLX inhibitor treatment in a dose-dependent manner but was abolished by Lon overexpression (Fig. 7B), suggesting that cisplatin-induced apoptosis is increased by the inhibition of mitochondria Ca^2+^ efflux and Lon overexpression is able to rescue the increased apoptosis. Overall, these results indicate that Lon overexpression allows cells to evade cell death under cisplatin treatment by activating cytosol calcium signaling via mitochondrial calcium release. To validate Lon-induced cisplatin resistance is through activation of NCLX, we used CGP37157 to check viability of Lon-overexpressing cells towards cisplatin using cell viability assay. We treated Lon-overexpressing cells and control cells with cisplatin and with or without NCLX inhibitor. We found that Lon-overexpressing cells increase the resistance towards cisplatin, whereas NCLX inhibition sensitized the death of Lon-overexpressing cells towards cisplatin (Fig. 7C). To validate Lon-induced resistance in tumor is through activation of NCLX, we used CGP37157 to check tumorigenicity of Lon-overexpressing cells in vivo using animal model. The Lon overexpression group significantly increased the tumor growth compared with the control group; the treatment of CGP37157 group inhibited Lon-induced tumor growth (Fig. 7D), suggesting that the CGP37157 treatment is able to overcome the drug resistance after prolonged cisplatin treatment. Therefore, we designed a set of in vivo experiment and examined the effect of prolonged cisplatin treatment and CGP37157 on the drug resistance (Fig. 7E). After the pretreatment of cisplatin at Day 10 and 12, the tumor size showed a rising trend after Day 14, and we continued different combination treatment after Day 26 for another four rounds of treatment. We found that cisplatin treatment groups failed to reduce the tumor size compared with after Day 26 (Fig. 7F); only the treatment of CGP37157 group reduced tumor size, confirming that the CGP37157 treatment is able to overcome the drug resistance after prolonged cisplatin treatment under Lon overexpression. These results confirm that Lon binds NCLX and induces mitochondrial calcium release to cytosol, which contributes to PYK2-SRC-STAT3 signaling-dependent cisplatin resistance. To associate the clinical significance of Lon- NCLX interaction in the drug resistance and cancer progression, we examined whether the expression of NCLX and Lon is clinically relevant in oral cancer. The expression pattern of NCLX and Lon in 6 samples of tumor tissues from OSCC patients was determined by immunohistochemistry (IHC) analysis. The clinicopathological characteristics of the patients in this study are as described in our previous results [3]. Representative samples of NCLX and Lon expression in the same OSCC patient are shown (Fig. 7G). The result showed that the expression pattern of NCLX is almost the same as the pattern of Lon in the tissues from OSCC patients (Fig. 7G), suggesting that a correlation between NCLX and Lon expression in OSCC patients.

## Discussion

Mitochondria are emerging as important organelles in cancer progression, metastasis, and chemotherapy resistance due to their dynamic bioenergetic plasticity and stress response pathways [1]. Mitochondria possess matrix proteases/chaperones to maintain protein quality control in stress conditions, oxidative and hypoxic stresses [43, 44]. For example, ClpP protease in mitochondria has been reported to involve in cisplatin resistance by activating efflux pumps to eliminate cisplatin from cells [45]. Mitochondrial Lon plays a central role in protein quality control, metabolism, and stress response, which maintains the normal function, biogenesis, and homeostasis of mitochondria [2, 8]. Lon upregulation is required for cancer cell survival and tumorigenesis after stress responses [12, 42]. Here our data show that cisplatin treatment causes mtDNA damage (adducts) that produces mitochondrial ROS and also upregulates Lon expression. We showed that Lon upregulation leads to resistance to cisplatin and Lon downregulation sensitizes cells towards cisplatin. Intriguingly, mitochondrial Lon contributes to cisplatin resistance through the interaction with NCLX, which induces an increase in cytosolic calcium level leading to inhibition of apoptosis (Fig. 7H). This study signifies that cisplatin-induced oxidative stress triggers upregulation of Lon protein that regulates calcium level in cancer cells. To our knowledge, this is the first work to report the role of Lon in the regulation of mitochondrial calcium.

Calcium (Ca^2+^) as a secondary messenger accounts for cell signaling, invasion, and metastasis to decide the cell fate of cancer cells [46]. Mitochondria sense Ca^2+^ signals and modulate cell fate and survival by regulating calcium signaling [24, 47, 48] via proteins like NCLX, which is a Na^+^/Ca^2+^ exchanger effluxing Ca^2+^ [27, 28, 49]. A study stated that NCLX knock-down in adrenergic cells causes accumulation of calcium in mitochondria and leads to mPTP opening and cell death [50]. Here we show that the resistance mechanism to cisplatin is involved in Lon-NCLX interaction that inhibits the accumulation of calcium in mitochondria preventing mPTP opening and cell death. Excess mitochondrial calcium triggers cell death therefore calcium homeostasis is a key phenomenon in cancer [27, 51]. For example, the results from mitochondrial proteomics of cisplatin-resistant cells showed high expression of NCLX, which hints involvement of increased Ca^2+^ efflux in preventing cell death [37]. We found that NCLX expression correlates with Lon expression, and Lon interacts with and thereby stabilizes NCLX to decrease mitochondrial calcium that induces cell death. Indeed, when we used CGP37157, a specific inhibitor of NCLX, to inhibit mitochondrial calcium release and found that CGP37157 inhibits cisplatin-induced calcium signaling and activates mitochondrial apoptosis. Here we report the importance of Lon-NCLX interaction in the cisplatin resistance of cancer cells via inducing calcium efflux from mitochondria to cytosol.

We observed that the Lon-NCLX axis increases cytosolic calcium level to trigger calcium-dependent PYK2-SRC-STAT3 signaling that induces Bcl-2 preventing cisplatin-induced apoptosis. Indeed, pharmacological inhibition of SRC abrogated cisplatin-induced apoptosis. We also indicated that Lon overexpression increases the level of IL-6. It is well known that exposure of cancer cells to chemotherapy leads to an increase of cancer stem cells that causes the resistance.

Our results suggested that Lon-induced Ca^2+^-dependent cisplatin resistance is involved in chemotherapy-induced cancer stemness, which is consistent with the reported roles of PYK2, SRC, and STAT3/IL-6 in the enrichment of cancer stem cells [32, 52]. Since the endoplasmic reticulum (ER) is an important source of calcium and the ER-Mitochondria contact sites (MAM) regulate calcium transfer to mitochondria, chemotherapy is able to increase MAM number and the calcium transfer from ER into mitochondria leading to cell death. However, cancer cells will induce the expression of certain antiapoptotic proteins like Bcl-2 located in MAM sites to decrease the transfer of calcium into mitochondria upon cisplatin treatment [31]. Indeed, our data show that increased Lon upregulates Bcl-2 protein expression upon cisplatin treatment leading to a decrease in apoptosis. Recently, Lon was found to be in MAM fraction under ER/mitochondria stress [53], but its functional role in MAM is yet to be established. The resistance to cisplatin by Lon in MAM may be mediated by decreasing calcium transfer from ER into mitochondria, which will be addressed in our future work.

In summary, we identified and validated the role of Lon in retrograde signaling on mtDNA damage and cisplatin resistance. This study for the first time addressed that mitochondrial Lon is involved in cisplatin resistance by regulating calcium signaling. We showed that Lon as a chaperone protein interacts with and stabilizes Na^+^/Ca^2+^ exchanger NCLX to release mitochondrial calcium into the cytosol. NCLX-mediated calcium release activates cytosolic calcium signaling PYK2-SRC-STAT3 signaling to activate downstream target genes, Bcl-2, and IL-6, to confer cisplatin resistance. In the future, mitochondrial Lon and NCLX inhibitors could act as adjuncts with cisplatin treatment to evade chemoresistance and improve the clinical outcome of oral cancer patients.

## Supplementary information

Supplementary material

Original Data File

aj-checklist

## Data Availability

All data needed to evaluate the conclusions of the paper are present in the main text and supplemental material. Data sharing not applicable as no datasets generated and/or analyzed for this study. Additional data related to this paper may be requested from the corresponding author.
