# Supplementary material for: Lon upregulation contributes to cisplatin resistance by triggering NCLX-mediated mitochondrial Ca2+ release in cancer cells
Source: Cell Death Dis. 2022 Mar 16;13(3):241. doi: 10.1038/s41419-022-04668-1 (PMC8927349; doi:10.1038/s41419-022-04668-1)
Supplement: Supplementary file 1 — Supplementary material [file 41419_2022_4668_MOESM1_ESM.docx]

**Lon upregulation contributes to cisplatin resistance by triggering NCLX-mediated mitochondrial Ca^2+^ release in cancer cells**

Vidhya Tangeda^1,2,3^, Yu Kang Lo^2^, Ananth Ponneri Babu Harisankar^1,2,3^, Han-Yu Chou^2^, Cheng-Liang Kuo^2^, Yung Hsi Kao^3^*, Alan Yueh-Luen Lee^1,2,3,4^* and Jang-Yang Chang^5^*

*: Corresponding author:

National Institute of Cancer Research, National Health Research Institutes, 35 Keyan Road, Zhunan, Miaoli 35053, Taiwan

Tel.: +886-37-206-166 ext. 31705

Fax: +886-37-586-463

E-mail: alanylee@nhri.edu.tw

ORCID ID: 0000-0003-0252-0571


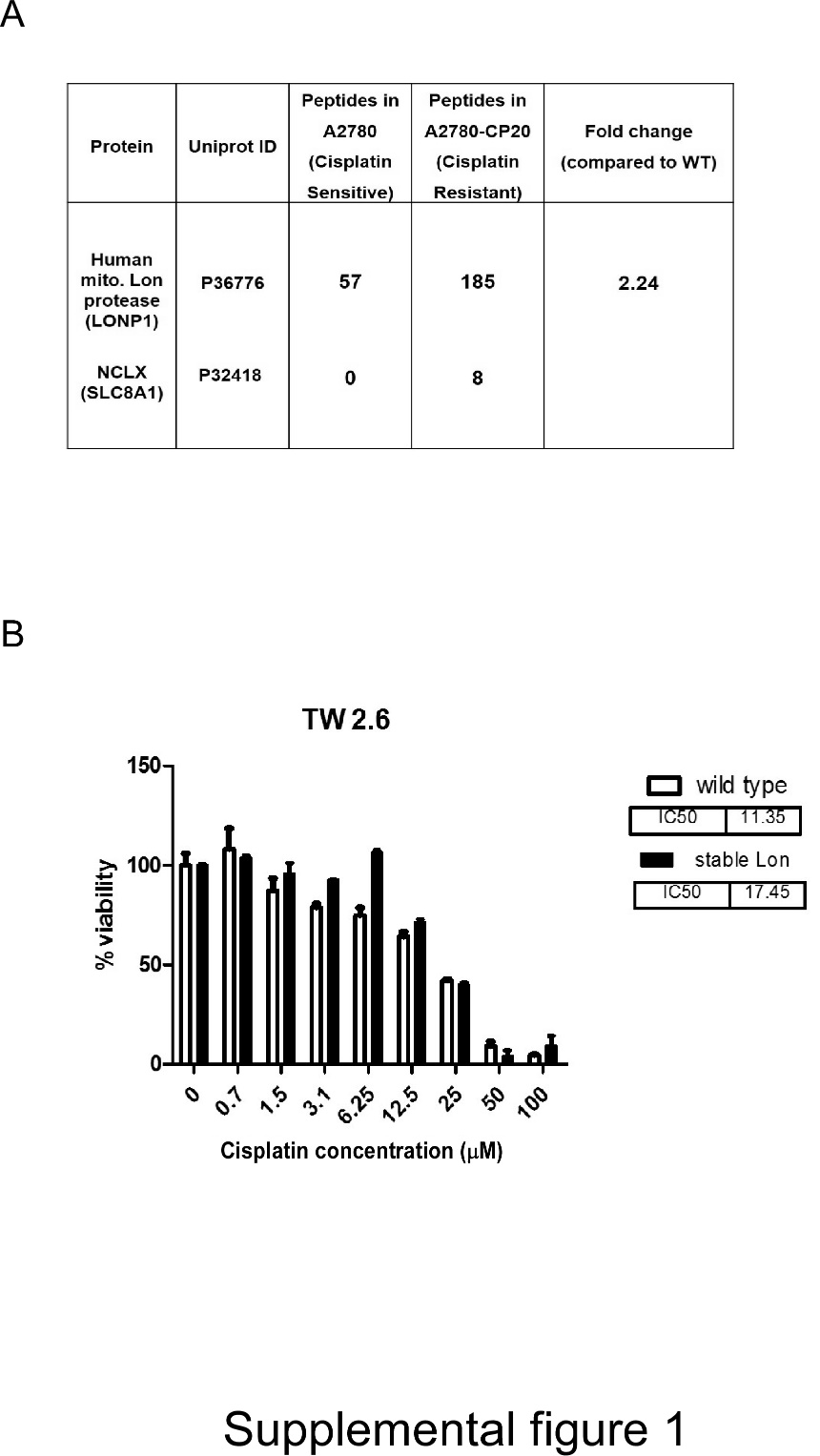


**Figure S1. Lon overexpression increases the viability of oral cancer cells upon cisplatin treatment**

A. Analysis of Lon and NCLX expression in the mitochondrial proteome dataset from cisplatin-resistant and -sensitive ovarian cancer cells.

B. Cisplatin cytotoxicity was assayed in TW2.6 cells overexpressing Lon or not following cisplatin treatment at different concentrations for 48 hrs. Cell cytotoxicity was performed using MTS assay. IC50 values were measured by using Log values using Graph Pad Prism 5.0 software.


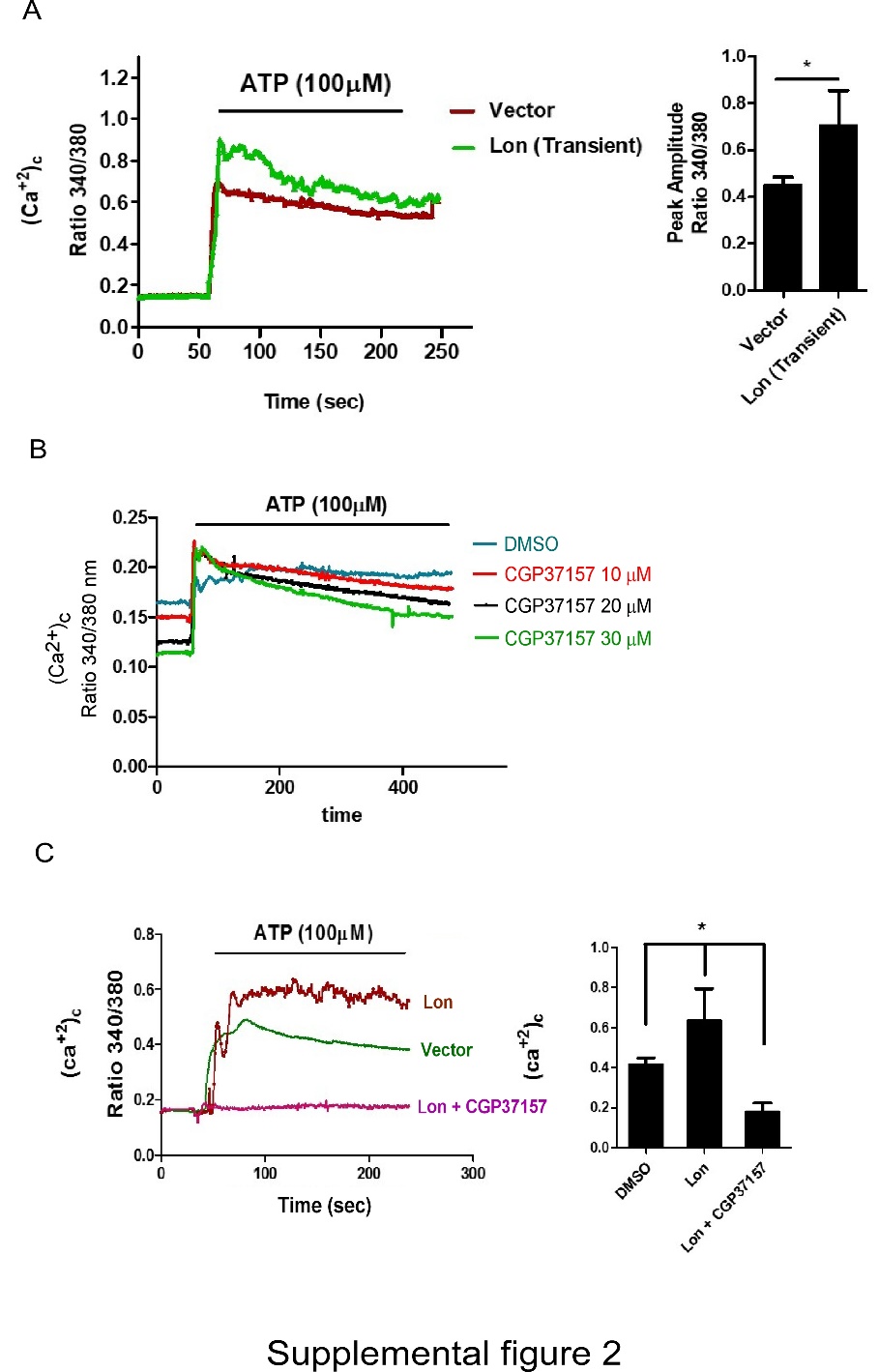


**Figure S2. Mitochondrial Lon increases cytosolic Ca2+ level through NCLX activity**

**A.** OEC-M1 cells were transfected with or without Lon plasmids and cytosolic calcium were stimulated with ATP and measured using Fura-2 AM dye (1 μM). Representative plot of the intensity of fluorescent images was shown.

**B.** OEC-M1 cells were treated with CGP37157 at indicated concentrations and cytosolic calcium were stimulated with ATP and measured using Fura-2 AM dye (1 μM). Representative plot of the intensity of fluorescent images was shown.

**C.** OEC-M1 cells transfected with or without Lon plasmids were treated with CGP37157 (NCLX inhibitor, 10 μM) or not and cytosolic calcium were stimulated with ATP and measured using Fura-2 AM dye (1 μM). Representative plot of the intensity of fluorescent images was shown. The error bars shown in the right panel represent the standard deviation from three independent experiments. *p<0.05.


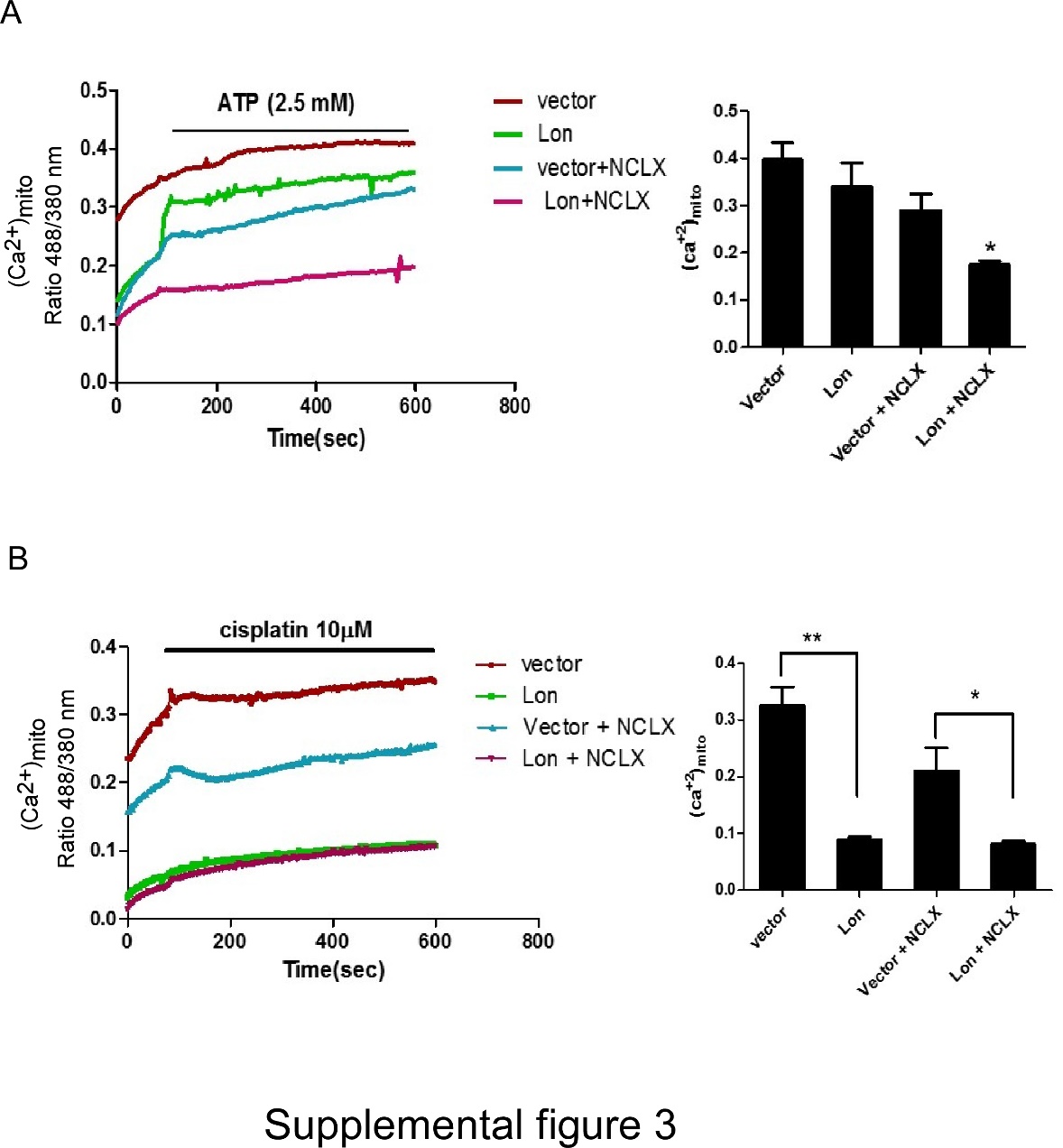


**Figure S3. Mitochondrial Lon decreases mitochondria Ca2+ level through NCLX activity**

**A.** OEC-M1 cells were co-transfected with Lon and/or NCLX plasmids and mitochondrial calcium were stimulated with ATP and measured by using mt-Pericam. Representative plot of the intensity of fluorescent images was shown. The error bars shown in the right panel represent the standard deviation from three independent experiments. *p<0.05.

**B.** OEC-M1 cells were co-transfected with Lon and/or NCLX plasmids and mitochondrial calcium were stimulated with cisplatin and measured by using mt-Pericam. Representative plot of the intensity of fluorescent images was shown. The error bars shown in the right panel represent the standard deviation from three independent experiments. *p<0.05; ** p < 0.01.
