# Supplementary material for: Lon upregulation contributes to cisplatin resistance by triggering NCLX-mediated mitochondrial Ca2+ release in cancer cells
Source: Cell Death Dis. 2022 Mar 16;13(3):241. doi: 10.1038/s41419-022-04668-1 (PMC8927349; doi:10.1038/s41419-022-04668-1)

Original Data of MS # CDDIS-21-2905

Fig. 1A

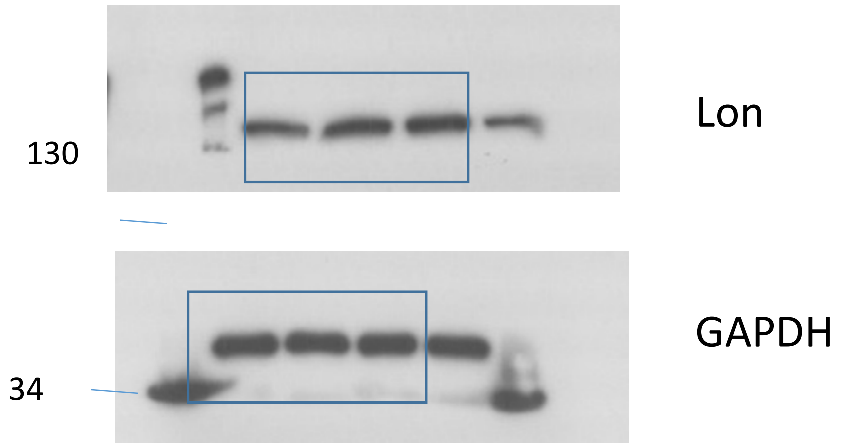

Fig. 1B

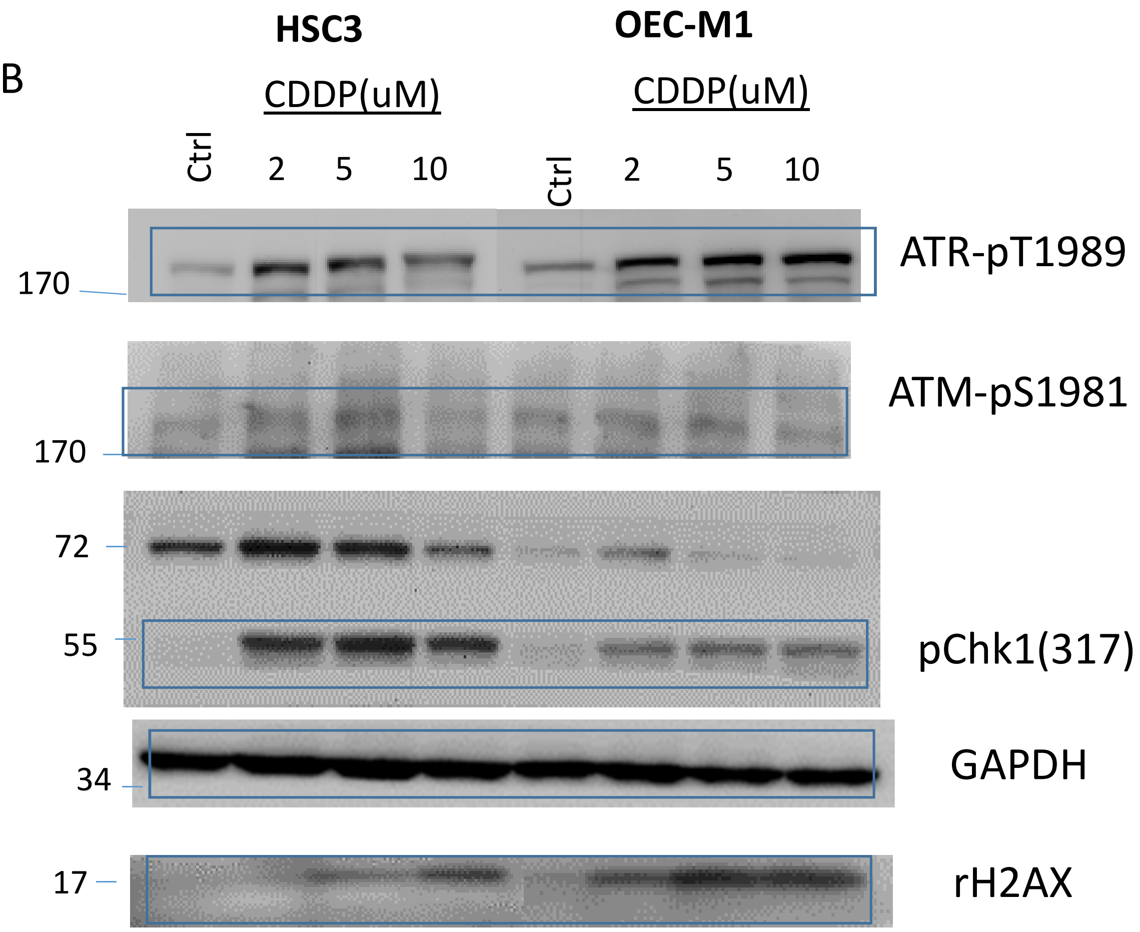

Fig. 2B

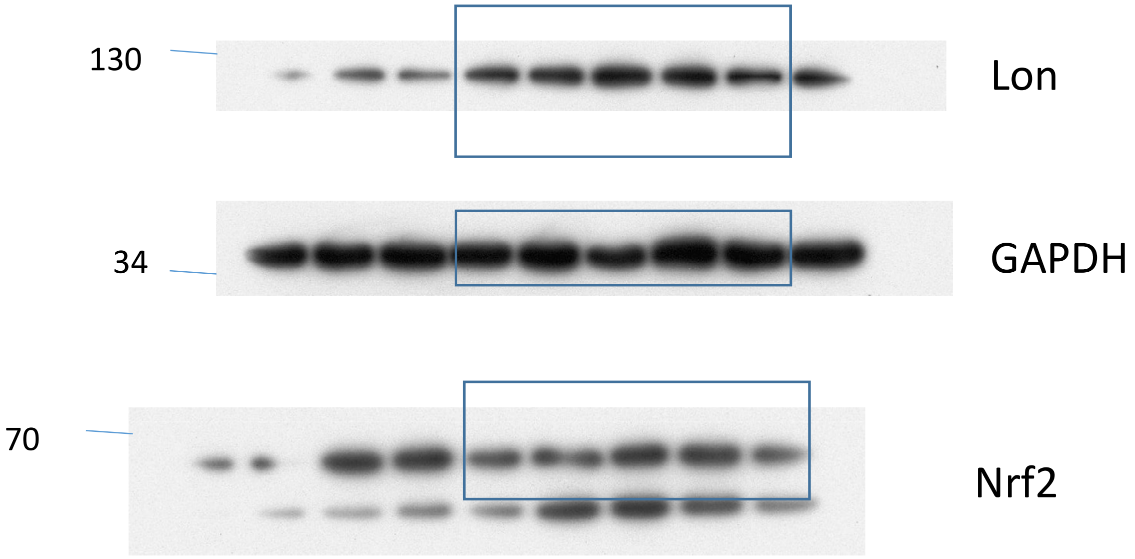

Fig. 2C

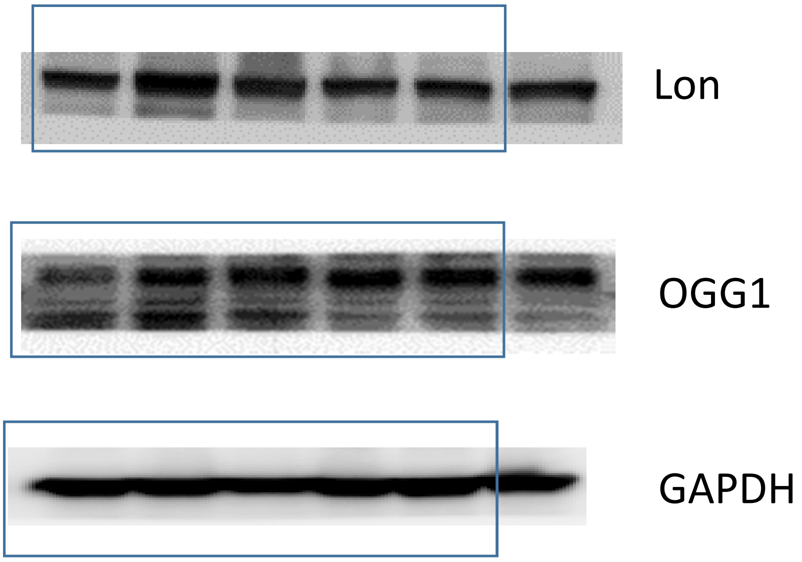

Fig. 3B

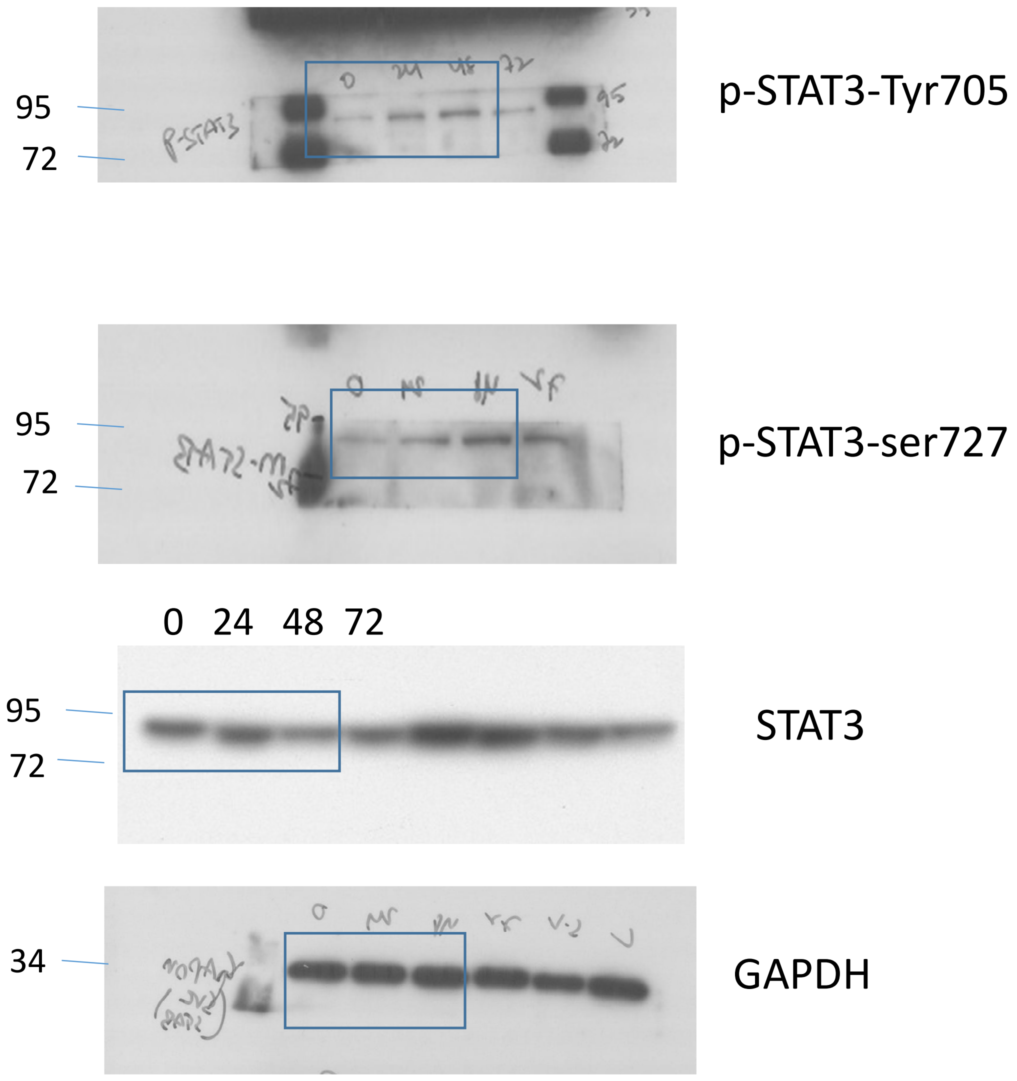
95
72
p-STAT3-Tyr705
95
72
p-STAT3-ser727
0 24 48 72
95
72
STAT3
34
GAPDH

Fig. 3C

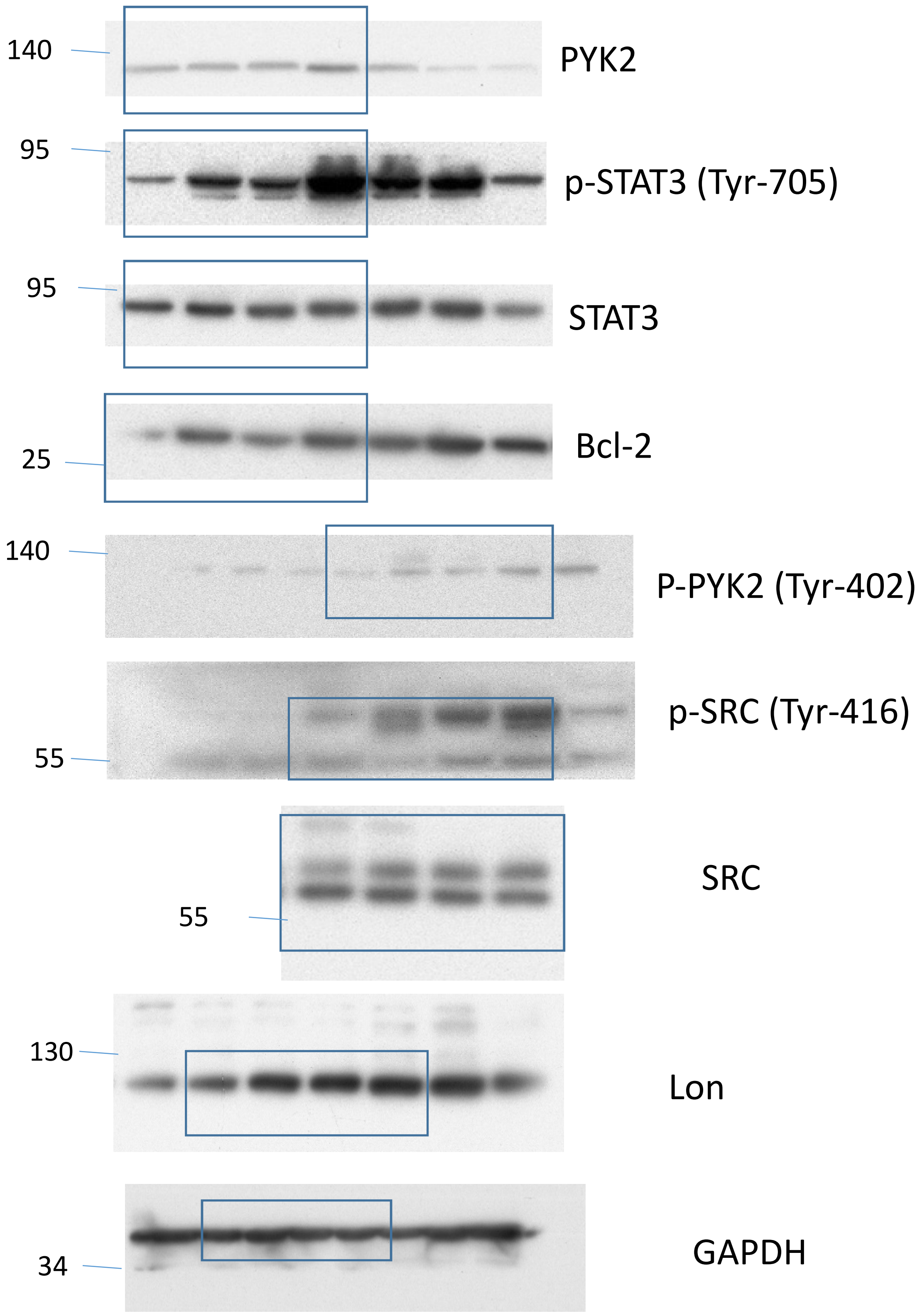

140
PYK2
95
p-STAT3 (Tyr-705)
95
STAT3
25
Bcl-2
140
P-PYK2 (Tyr-402)
55
p-SRC (Tyr-416)
55
SRC
130
Lon
34
GAPDH

Fig. 3D

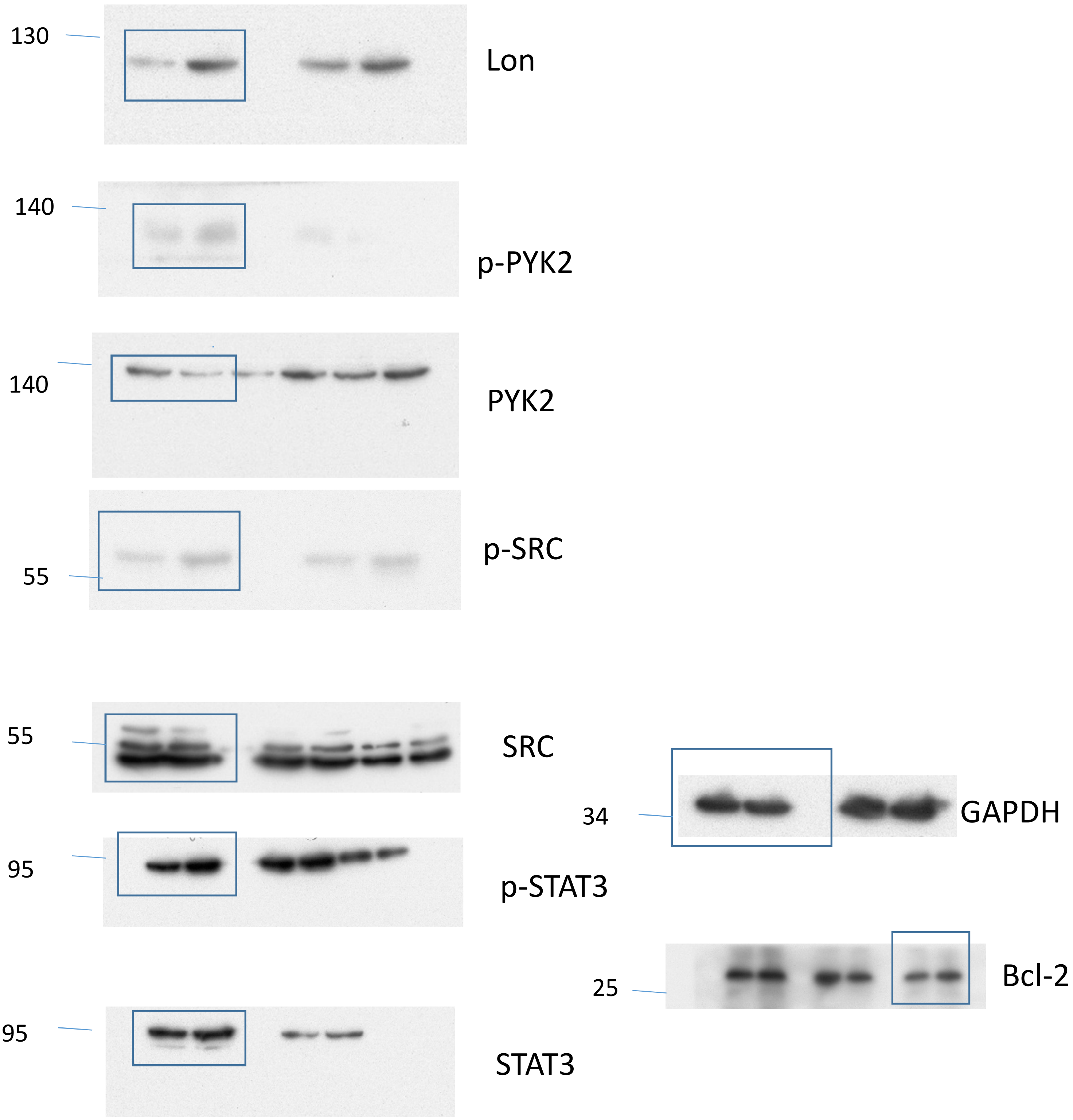
130
Lon
140
p-PYK2
140
PYK2
p-SRC
55
55
SRC
34
GAPDH
95
p-STAT3
25
Bcl-2
95
STAT3

Fig.3D

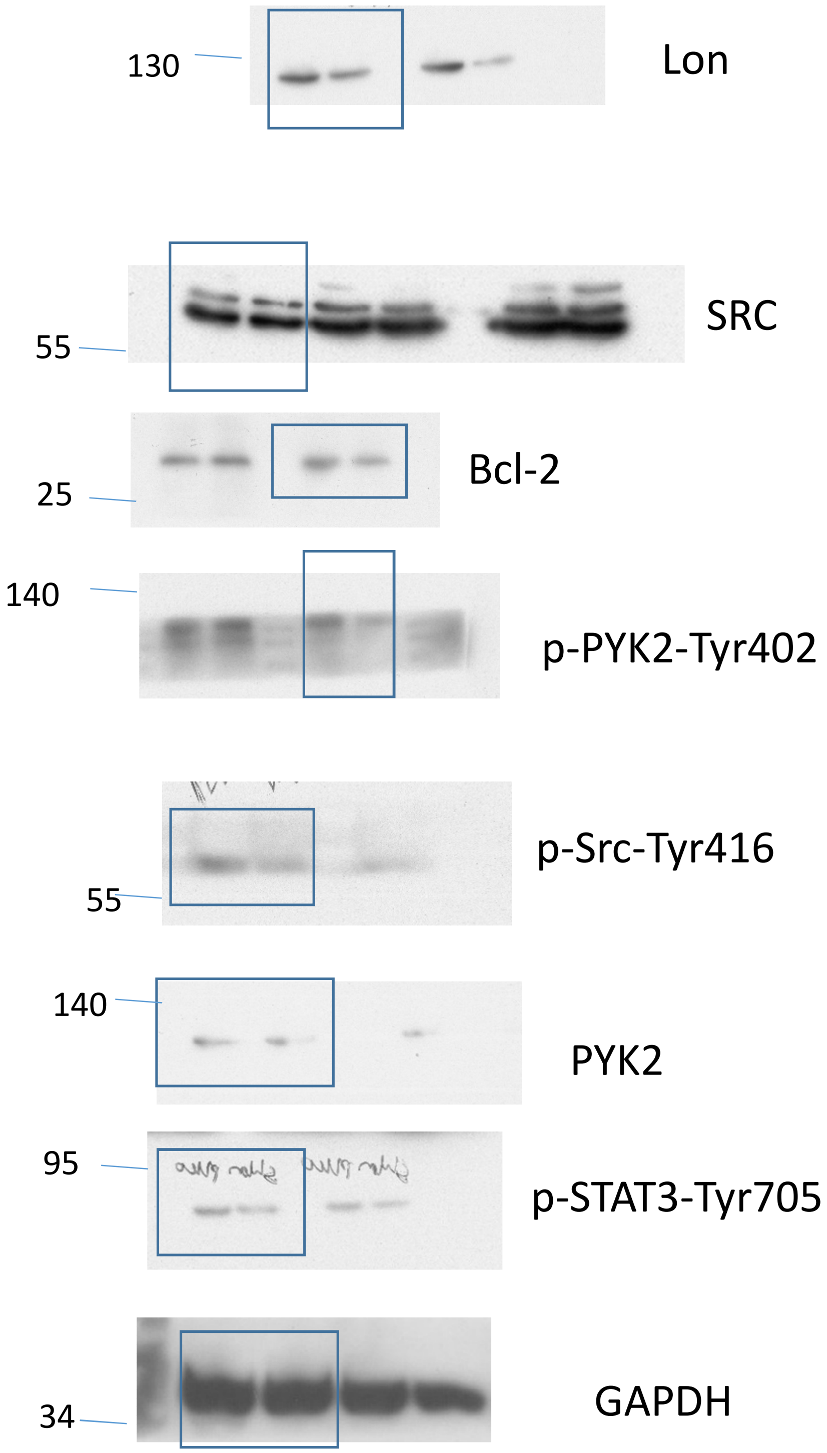
130
Lon
SRC
55
Bcl-2
25
140
p-PYK2-Tyr402
p-Src-Tyr416
55
140
PYK2
95
p-STAT3-Tyr705
34
GAPDH

Fig. 3E

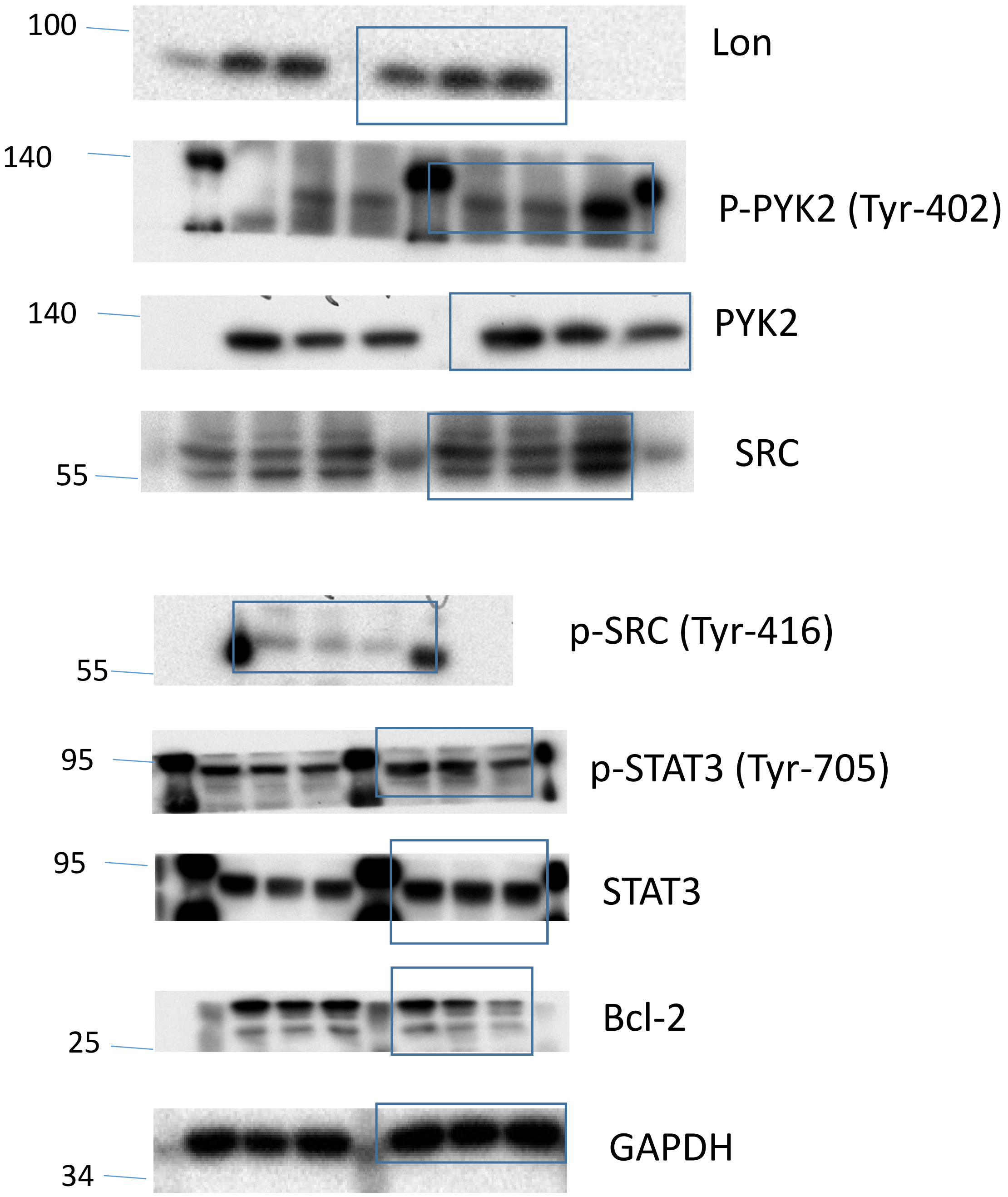
100
Lon
140
P-PYK2 (Tyr-402)
140
PYK2
55
SRC
55
p-SRC (Tyr-416)
95
p-STAT3 (Tyr-705)
95
STAT3
25
Bcl-2
34
GAPDH

Fig. 6A

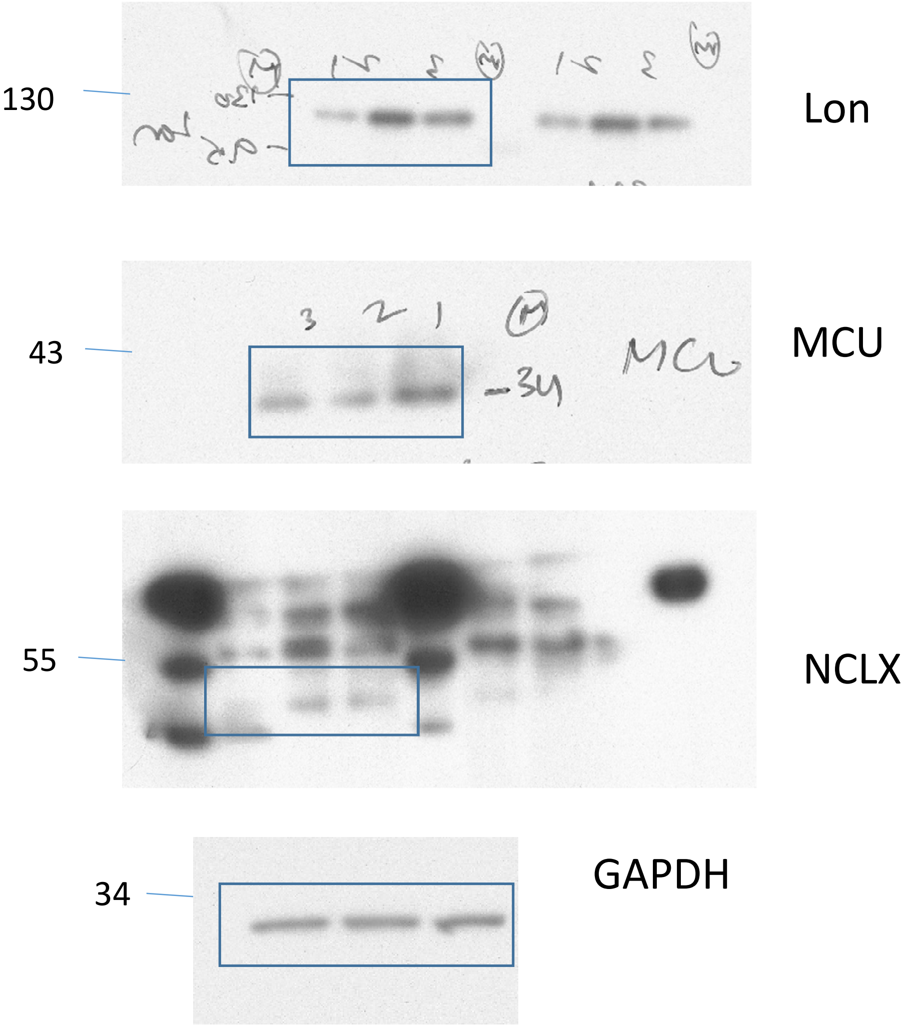

Fig. 6B

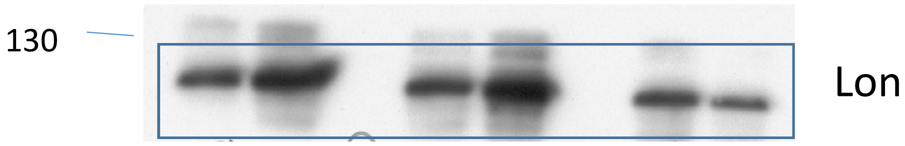

Fig. 6C

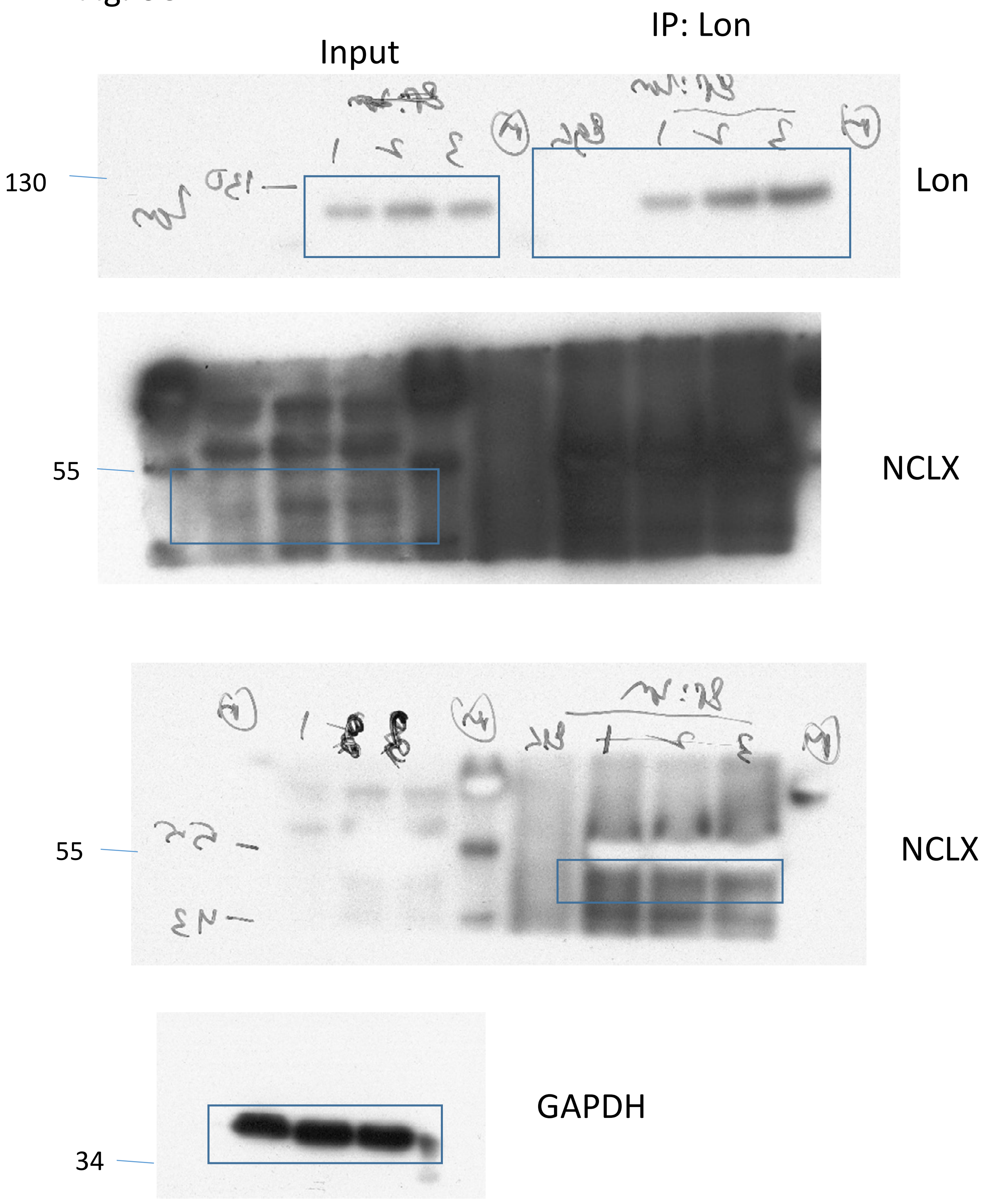

Fig. 6C

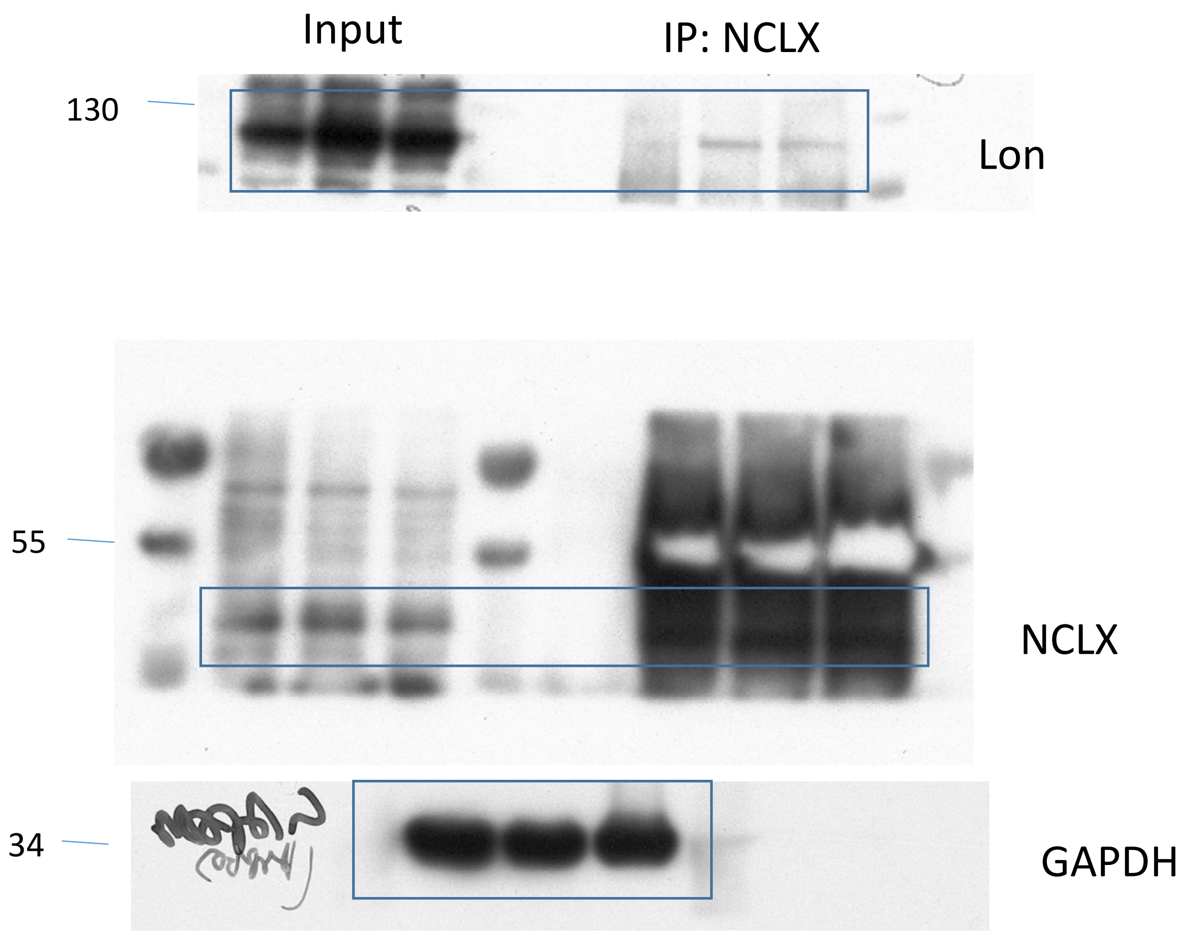

Fig 6D

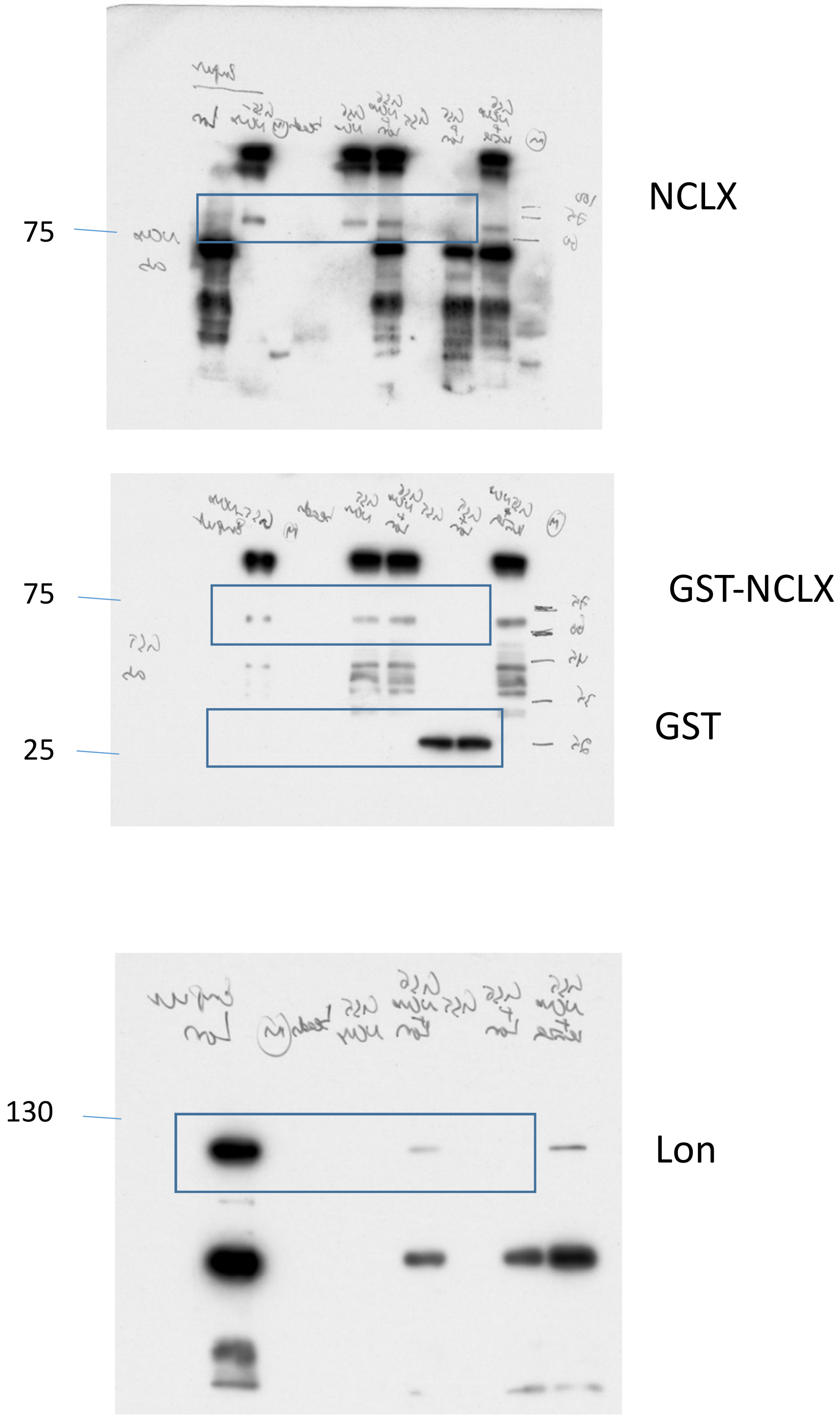

Fig. 6F

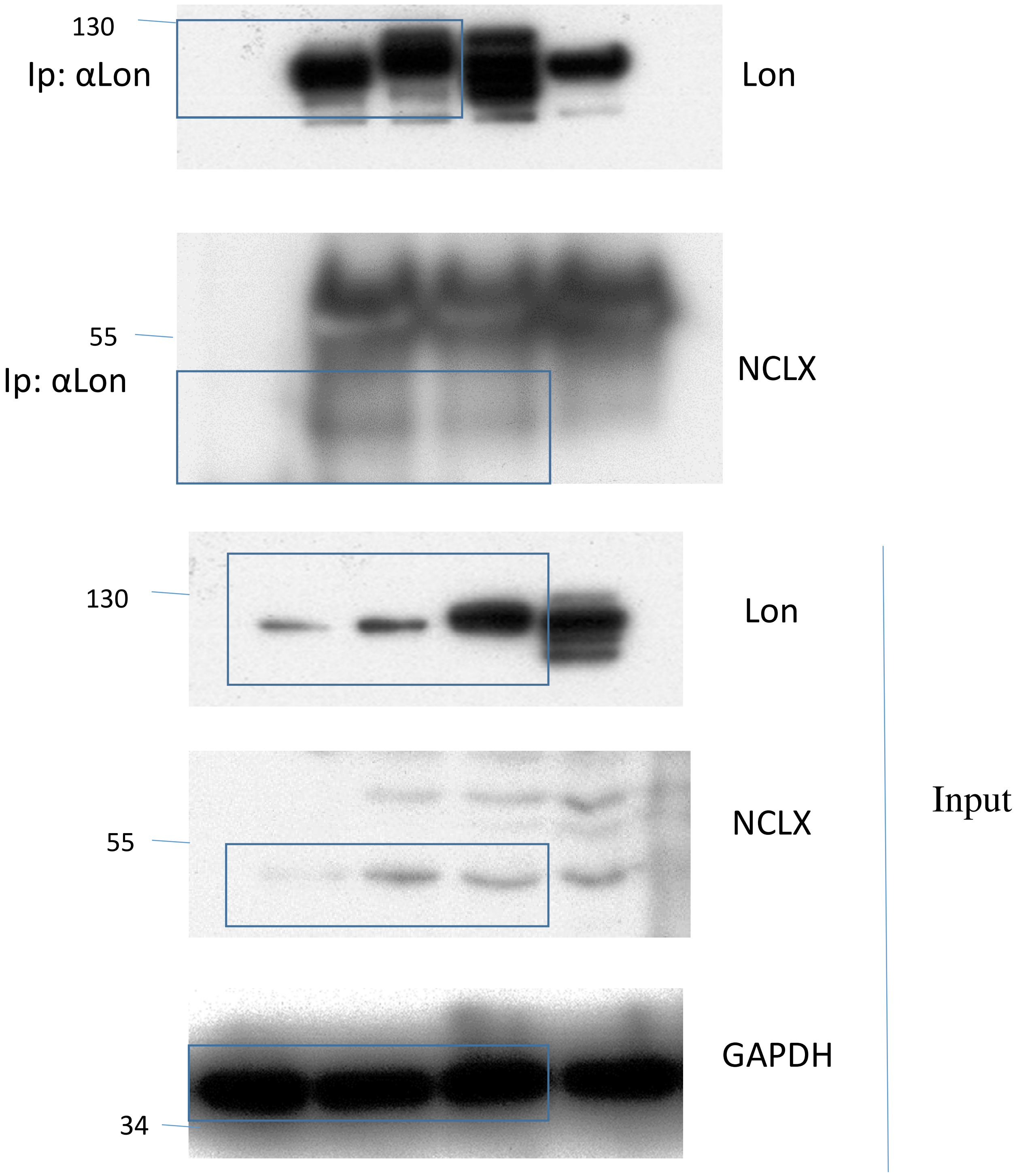

Fig. 7B

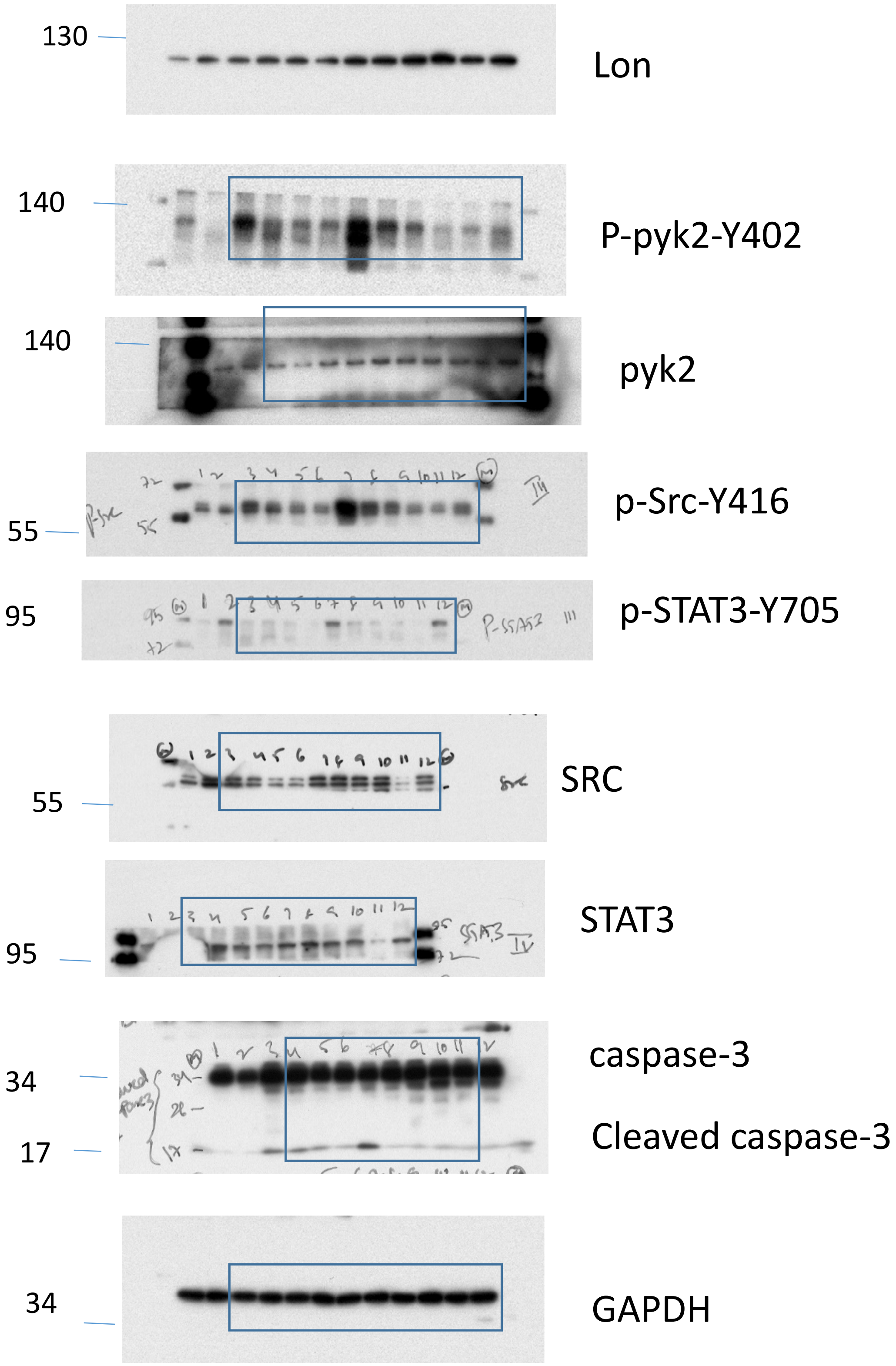
130
Lon
140
P-pyk2-Y402
140
pyk2
55
p-Src-Y416
95
p-STAT3-Y705
55
SRC
95
STAT3
34
17
caspase-3
Cleaved caspase-3
34
GAPDH

Fig. 2D

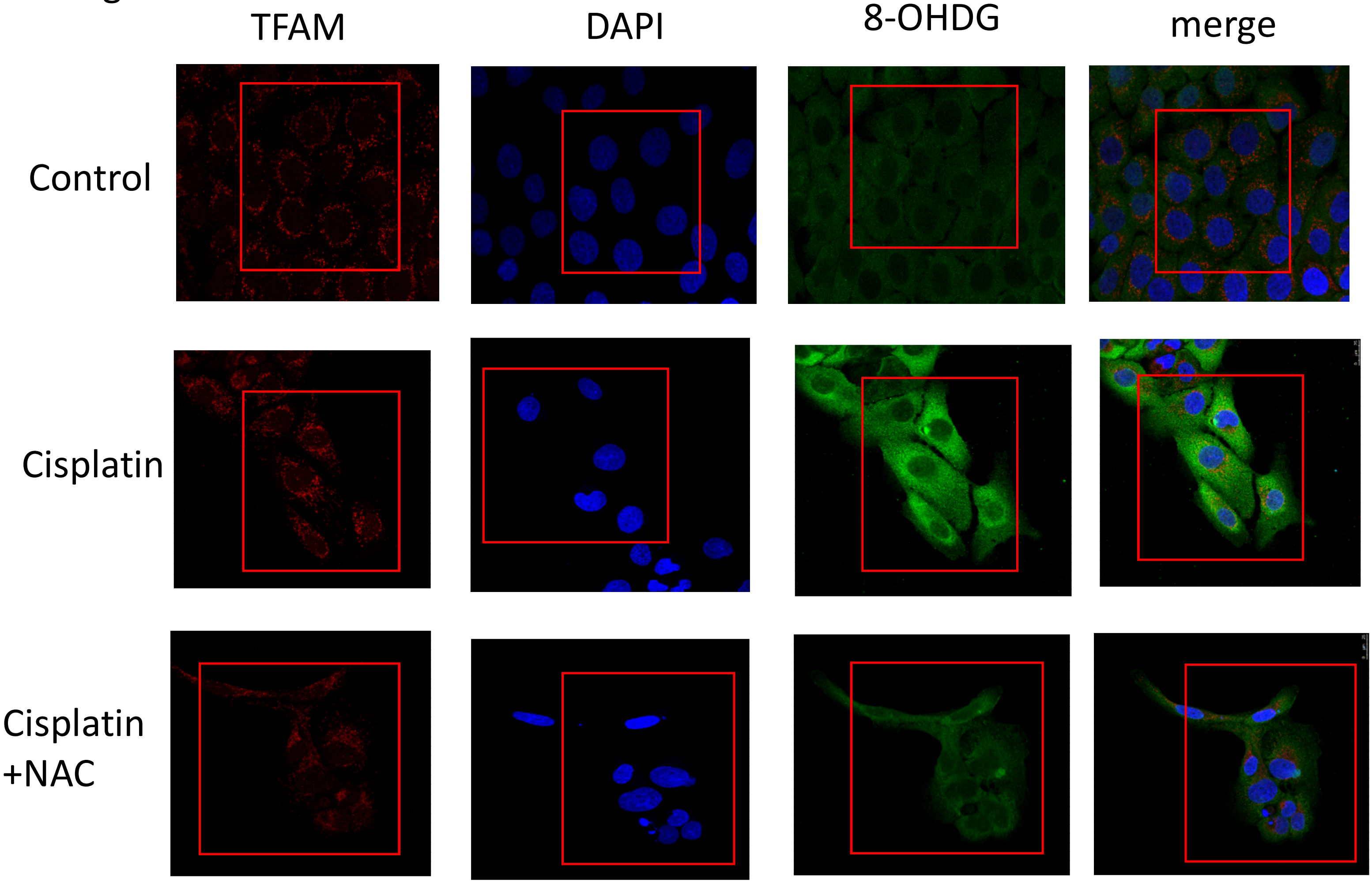

Fig. 6E

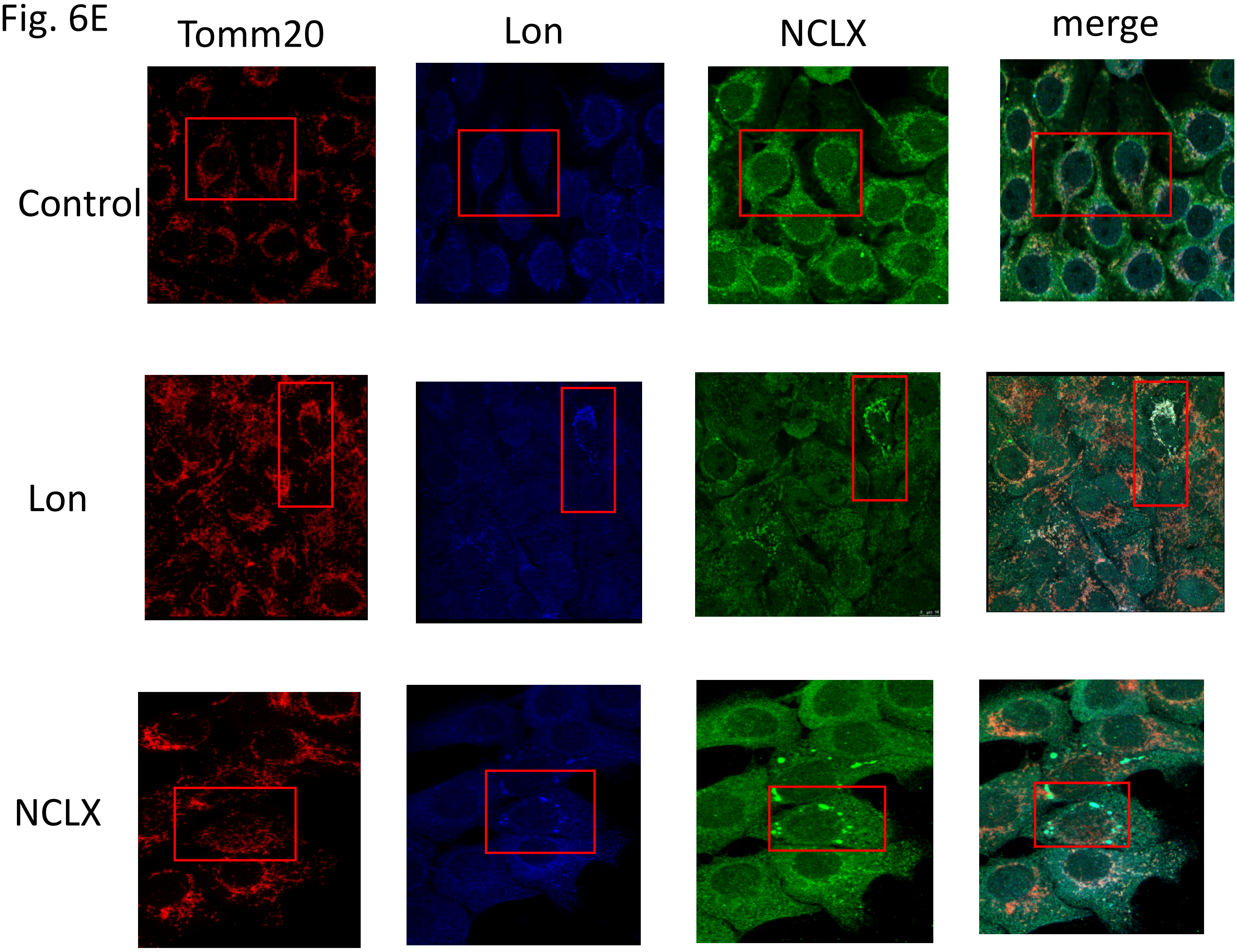

Supplement: Supplementary file 2 — Original Data File [file 41419_2022_4668_MOESM2_ESM.pdf]
